# Dynamic modeling of cis-regulatory circuits and gene expression prediction via cross-gene identification

**DOI:** 10.1186/1471-2105-6-258

**Published:** 2005-10-18

**Authors:** Li-Hsieh Lin, Hsiao-Ching Lee, Wen-Hsiung Li, Bor-Sen Chen

**Affiliations:** 1 Lab. of System Biology, National Tsing Hua University, 101, Sec 2, Kuang Fu Road, Hsinchu, 300, Taiwan.; 2 Department of Life Science & Institute of Bioinformatics and Structural Biology, National Tsing Hua University, Hsinchu, 300, Taiwan.; 3 Department of Ecology and Evolution, University of Chicago, USA.; 4 Genomics Research Center, Academia Sinica, Taipei, Taiwan.

## Abstract

**Background:**

Gene expression programs depend on recognition of *cis *elements in promoter region of target genes by transcription factors (TFs), but how TFs regulate gene expression via recognition of *cis *elements is still not clear. To study this issue, we define the *cis*-regulatory circuit of a gene as a system that consists of its *cis *elements and the interactions among their recognizing TFs and develop a dynamic model to study the functional architecture and dynamics of the circuit. This is in contrast to traditional approaches where a *cis*-regulatory circuit is constructed by a mutagenesis or motif-deletion scheme. We estimate the regulatory functions of *cis*-regulatory circuits using microarray data.

**Results:**

A novel cross-gene identification scheme is proposed to infer how multiple TFs coordinate to regulate gene transcription in the yeast cell cycle and to uncover hidden regulatory functions of a *cis*-regulatory circuit. Some advantages of this approach over most current methods are that it is based on data obtained from intact *cis*-regulatory circuits and that a dynamic model can quantitatively characterize the regulatory function of each TF and the interactions among the TFs. Our method may also be applicable to other genes if their expression profiles have been examined for a sufficiently long time.

**Conclusion:**

In this study, we have developed a dynamic model to reconstruct *cis*-regulatory circuits and a cross-gene identification scheme to estimate the regulatory functions of the TFs that control the regulation of the genes under study. We have applied this method to cell cycle genes because the available expression profiles for these genes are long enough. Our method not only can quantify the regulatory strengths and synergy of the TFs but also can predict the expression profile of any gene having a subset of the *cis *elements studied.

## Background

*Cis*-regulatory circuits have been applied to model cell cycle control and other developmental processes [1,2]. Recently, the genetic regulatory and transcriptional networks of yeast have been studied [3-8]. However, in order to understand the regulation of any particular gene in the network, one should study carefully how the genes in the network collectively operate with remarkable precision in response to environmental cues and the structure and function of the *cis*-regulatory circuit of the gene [7]. The *cis*-regulatory circuit of a gene consists of its *cis *elements, i.e., binding motifs of transcription factor (TF), and the interactions among their recognizing TFs. The *cis *elements of a gene can be considered as the information processing units in the regulatory circuit; they receive multiple inputs from the TFs that bind the *cis *elements of the gene. The output is the instruction for the transcription apparatus to determine whether the gene is to be expressed at a specific rate or to be repressed [9,10].

A *cis*-regulatory circuit may be regarded as a control device that is called into play by the TFs that have target sites inside the promoter [14-20]. A well-known example is the promoter region of the developmentally regulated *endo *16 gene of the sea urchin [12-14]. It is about ~2300 bp in length and consists of several clusters of target sites for distinct functions. Yuh *et al. *[12-14] have explored the function of each subregion of the *endo *16 system and every target site within each subregion, using a *cis*-regulatory logic model.

However, a drawback of most current methods for inferring *cis*-regulatory circuits is that they rely on changing or deleting some binding site sequences (e.g., [12-14]), which may not provide intact functional information for reconstructing the *cis*-regulatory circuit. The deletion or mutation experiments may change or destroy the original *cis*-regulatory circuit structure. Using such data, one may lose significant interactions among transcription factors (TFs). Obviously, it is appealing to develop a method that can infer intact *cis*-regulatory circuits. Recently, there are some statistical and system level approaches to study the genome-wide transcription regulation and address cooperativity among TFs ([7-11,15,16]). Important advances have been made toward understanding transcriptional regulatory networks. One strategy infers global networks directly from whole genome microarray data, and another strategy focuses on the identification of shared *cis *elements in the promoters of co-regulated genes, signified by similar expression profiles [8]. In this paper we develop a new method to combine microarray data and TF-binding location data by chromatin immunoprecipitation [5,18] to study the regulatory and interaction functions of various *cis *elements with regard to the target gene. The data of Lee *et al. *2002 [5] can reveal the *in vivo *physical interactions of TFs with their *cis *elements on the promoter and therefore can provide a more reliable view of functional interactions between TFs and *cis *elements. Combining these types of data and microarray data, we propose a novel cross-gene identification scheme to infer how multiple TFs coordinate to regulate gene transcription. Our approach is rather different from most existing statistical and system level methods for analyzing gene expression data. Our results show that this novel method is suitable for deciphering the complex TFs interactions and for predicting the gene expression. In addition, we also identify the dynamic regulatory functions of TFs interaction in the yeast cell cycle, which cannot be achieved by current methods.

## Results

### Characterizing the *cis*-regulatory circuit of a gene

In this study, there are two steps for characterizing the *cis*-regulatory circuit of a gene. The first is to find a cluster of genes that includes the gene of interest and a number of other genes each of which shares a subset of *cis *elements with the gene of interest. Assuming a certain regulatory function for each of the TFs that recognize the *cis *elements of genes in the cluster and certain interaction functions among the TFs of a circuit, we set up dynamic equations for the *cis*-regulatory circuits of the genes in the cluster to describe their expression profiles. Since each gene in the cluster shares some *cis *elements with the gene of interest, a matrix of *cis *elements for the cluster of genes can be constructed. In this model, the regulatory functions of individual TFs and the interactions among TFs can be estimated from microarray data. In the second step, a cross-gene identification scheme is developed with an array of expression profiles of genes in the cluster (e.g., Spellman *et al.*, 1998 [18]) to identify regulatory functions of the TFs and their possible interactions for each gene in the cluster; the parameters are estimated by the least square estimation algorithm.

Finally, plugging these estimated regulatory functions and interactions into the dynamic equations, one can explicitly describe the *cis*-regulatory circuit of the gene of interest.

### Choice of a cluster of genes

**Figure 1:**
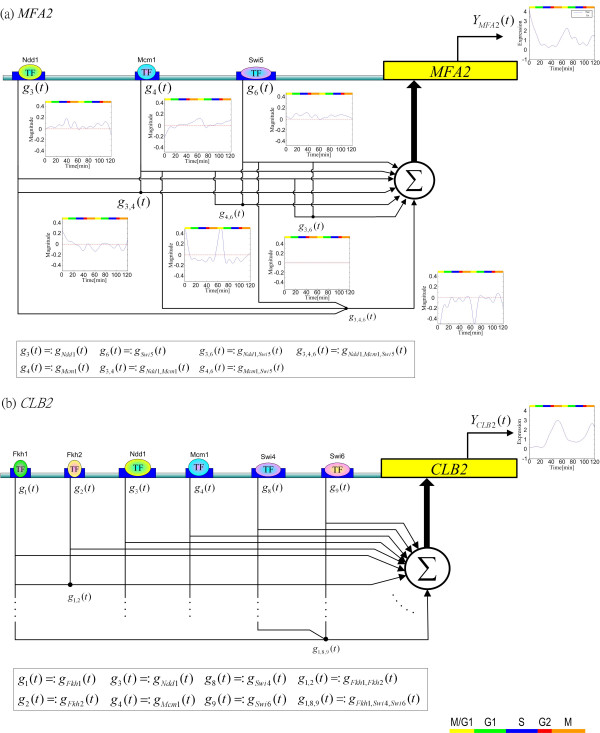
Dynamic model of the *cis*-regulatory circuit of gene *MFA2 *(a) and of gene *CLB2 *(b). The genome-wide TF-binding location data obtained using chromatin immunoprecipitation [4] is used to identify the transcription factor binding motifs (*cis *elements). A binding transcription factor *p *has a regulatory function *g*_*p*_(*t*) and interacts with other recognizing TFs to produce the regulatory functions *g*_*p*,*q*_(*t*) and *g*_*p*,*q*,*r*_(*t*). These regulatory functions are the inputs of the *cis*-regulatory circuit and generate the dynamic output (i.e., the expression profile) of the target gene. Different phases of the cell cycle are indicated by the colored bar at the right lower corner.

As an illustration, suppose some genes of the yeast cell cycle are of interest. We find a cluster of genes for each gene of interest according to their *cis *elements found in Simon *et al. *2001 [4]. To simplify the analysis, we consider only the nine TFs that are currently known to be important cell cycle TFs of the yeast (i.e., Mbp1, Swi4, Swi6, Mcm1, Fkh1, Fkh2, Ndd1, Swi5, and Ace2). The cluster of genes for the gene of interest is called the reference gene cluster (RGC). In an RGC, we assume that each gene shares some *cis *elements of the gene of interest. Furthermore, the regulatory functions and the interactions of the TFs recognizing the same *cis *elements are assumed to be the same for all genes in the RGC. For example, in Figure 1a gene *MFA2 *is the gene of interest; it causes cell cycle arrest and is essential for mating in yeast [19]. This gene has three main *cis *elements, Ndd1, Mcm1 and Swi5 [4], from which we want to reconstruct the *cis*-regulatory circuit of *MFA2 *from yeast microarray data. The *cis *elements of *MFA2 *are denoted as follows:

*MFA2*:{Ndd1, Mcm1, Swi5}.     (1)

Some genes chosen for the cluster and their *cis *elements are:

*YAL022C*:{Swi5}, *YAR018C*:{Ndd1, Mcm1}, *YKL163W*:{Mcm1, Swi5} ....

The cluster of genes can be represented by a connectivity matrix of *cis *elements as shown in Table 1 in which "1" denotes the connection of a *cis *element with a gene, while "0" means no connection. Similarly, the RGC for gene *CLB2 *can be represented by the connectivity matrix in Table 2.

**Table 1:** The reference gene clusters (RGCs) of *MFA2*. Target Gene *MFA2 *and the connectivities to *cis *elements.

	Fkh1	Fkh2	Ndd1	Mcm1	Ace2	Swi5	Mbp1	Swi4	Swi6										
*MFA2 *(YNL145W)	0	0	1	1	0	1	0	0	0										

**Reference genes cluster (RGC) and their connectivities to *cis *elements**																			

ORF	Fkh1	Fkh2	Ndd1	Mcm1	Ace2	Swi5	Mbp1	Swi4	Swi6	ORF	Fkh1	Fkh2	Ndd1	Mcm1	Ace2	Swi5	Mbp1	Swi4	Swi6

YAL022C	0	0	0	0	0	1	0	0	0	YKL209C	0	0	0	1	0	0	0	0	0
YAR018C	0	0	1	1	0	0	0	0	0	YLR274W	0	0	0	1	0	0	0	0	0
YDR150W	0	0	1	0	0	0	0	0	0	YML050W	0	0	1	1	0	0	0	0	0
YFL026W	0	0	0	1	0	0	0	0	0	YML125C	0	0	0	0	0	1	0	0	0
YGL032C	0	0	0	1	0	0	0	0	0	YMR001C	0	0	1	0	0	0	0	0	0

YIL050W	0	0	0	0	0	1	0	0	0	YMR002W	0	0	1	0	0	0	0	0	0
YIL129C	0	0	0	0	0	1	0	0	0	YMR253C	0	0	0	1	0	0	0	0	0
YJL079C	0	0	1	1	0	0	0	0	0	YNL056W	0	0	1	1	0	0	0	0	0
YJL157C	0	0	0	1	0	0	0	0	0	YNL058C	0	0	1	1	0	0	0	0	0
YKL163W	0	0	0	1	0	1	0	0	0	YNL145W	0	0	1	1	0	1	0	0	0

YKL164C	0	0	0	1	0	1	0	0	0	YOR066W	0	0	0	1	0	0	0	0	0

**Table 2:** The reference gene clusters (RGCs) of *CLB2*. Target Gene *CLB2 *and the connectivities to *cis *elements.

	Fkh1	Fkh2	Ndd1	Mcm1	Ace2	Swi5	Mbp1	Swi4	Swi6										
*CLB2 *(YPR119W)	1	1	1	1	0	0	0	1	1										

**Reference genes cluster (RGC) and their connectivities to *cis *elements**																			

ORF	Fkh1	Fkh2	Ndd1	Mcm1	Ace2	Swi5	Mbp1	Swi4	Swi6	ORF	Fkh1	Fkh2	Ndd1	Mcm1	Ace2	Swi5	Mbp1	Swi4	Swi6
YAR018C	0	0	1	1	0	0	0	0	0	YKR013W	0	0	0	0	0	0	0	1	1
YBR133C	0	1	0	0	0	0	0	0	0	YLR056W	0	0	0	0	0	0	0	1	1
YBR138C	1	0	1	0	0	0	0	0	0	YLR084C	0	1	1	1	0	0	0	1	0
YBR139W	1	0	1	0	0	0	0	0	0	YLR131C	0	1	1	1	0	0	0	0	0
YCL063W	1	1	0	0	0	0	0	0	0	YLR190W	0	1	1	1	0	0	0	0	0

YDL227C	0	0	0	0	0	0	0	1	1	YLR209C	1	0	0	0	0	0	0	0	0
YDR033W	0	1	1	0	0	0	0	0	0	YLR210W	1	0	0	0	0	0	0	0	0
YDR146C	0	1	1	1	0	0	0	0	0	YLR274W	0	0	0	1	0	0	0	0	0
YDR150W	0	0	1	0	0	0	0	0	0	YLR342W	0	0	0	0	0	0	0	1	1
YDR224C	0	0	0	0	0	0	0	1	1	YML050W	0	0	1	1	0	0	0	0	0

YDR225W	0	0	0	0	0	0	0	1	1	YML064C	1	1	0	0	0	0	0	0	0
YDR507C	0	0	0	1	0	0	0	1	1	YMR001C	0	0	1	0	0	0	0	0	0
YEL017W	1	0	0	0	0	0	0	0	0	YMR002W	0	0	1	0	0	0	0	0	0
YEL040W	0	1	0	1	0	0	0	1	1	YMR015C	1	0	0	0	0	0	0	1	0
YER001W	0	0	0	0	0	0	0	1	1	YMR183C	1	0	0	0	0	0	0	0	0

YFL026W	0	0	0	1	0	0	0	0	0	YMR253C	0	0	0	1	0	0	0	0	0
YGL032C	0	0	0	1	0	0	0	0	0	YMR305C	0	0	0	0	0	0	0	1	1
YGL038C	0	0	0	0	0	0	0	1	1	YMR307W	0	0	0	0	0	0	0	1	1
YGL116W	0	1	1	1	0	0	0	0	0	YNL056W	0	0	1	1	0	0	0	0	0
YGR014W	0	0	0	0	0	0	0	1	1	YNL058C	0	0	1	1	0	0	0	0	0

YGR099W	1	0	0	0	0	0	0	0	0	YNL231C	0	1	0	0	0	0	0	1	1
YGR151C	0	0	0	0	0	0	0	1	0	YNL300W	0	0	0	0	0	0	0	1	1
YGR152C	0	0	0	0	0	0	0	1	0	YOL011W	0	0	0	0	0	0	0	1	0
YGR153W	0	0	0	0	0	0	0	1	0	YOL030W	1	0	0	0	0	0	0	0	0
YGR221C	0	0	0	0	0	0	0	1	1	YOL114C	0	0	0	0	0	0	0	1	1

YGR279C	0	0	0	0	0	0	0	1	1	YOR066W	0	0	0	1	0	0	0	0	0
YHR061C	0	1	0	0	0	0	0	1	1	YOR073W	0	1	0	0	0	0	0	0	0
YIL056W	0	1	1	0	0	0	0	1	1	YOR372C	0	0	0	0	0	0	0	1	1
YIL121W	0	0	0	0	0	0	0	1	0	YPL032C	1	0	0	0	0	0	0	0	0
YIL123W	0	1	0	1	0	0	0	1	1	YPL116W	1	0	0	0	0	0	0	0	0

YIL158W	0	1	1	1	0	0	0	0	0	YPL127C	0	0	0	0	0	0	0	1	1
YJL051W	0	1	1	1	0	0	0	0	0	YPL141C	1	0	0	0	0	0	0	0	0
YJL079C	0	0	1	1	0	0	0	0	0	YPL155C	0	1	0	0	0	0	0	0	0
YJL157C	0	0	0	1	0	0	0	0	0	YPL163C	0	0	0	0	0	0	0	1	1
YJL158C	0	1	1	0	0	0	0	1	1	YPL255W	0	0	0	0	0	0	0	0	1

YJR054W	0	0	0	0	0	0	0	1	1	YPL256C	0	0	0	0	0	0	0	0	1
YJR092W	1	1	1	1	0	0	0	0	0	YPR013C	1	0	0	0	0	0	0	1	0
YJR110W	0	1	0	0	0	0	0	0	0	YPR119W	1	1	1	1	0	0	0	1	1
YKL096W	0	1	0	0	0	0	0	1	1	YPR149W	0	1	1	0	0	0	0	1	0
YKL103C	0	0	0	0	0	0	0	1	0	YPR159W	0	0	0	0	0	0	0	1	1

YKL209C	0	0	0	1	0	0	0	0	0										

### Dynamic modeling of *cis*-regulatory circuits

Figure 1 illustrates the leaky integrator-based dynamic models of the *cis*-regulatory circuits of two yeast genes (*MFA2 *and *CLB2*) [1,2]. The dynamics of gene expression can be modeled by a simple first-order nonlinear differential equation that is well established and analyzed in [17]; each model includes the possible regulatory functions of the individual TFs and possible interactions among the TFs. For the target gene *MFA2 *(Figure 1a), the *cis*-regulatory circuit is modeled by the following dynamic equation

Y˙MFA2(t)=gNdd1(t)+gMcm1(t)+gSwi5(t)+gNdd1,Mcm1(t)+      gNdd1,Swi5(t)+gMcm1,Swi5(t)+gNdd1,Mcm1,Swi5(t)−λMFA2YMFA2(t)+εMFA2(t),     (2)
 MathType@MTEF@5@5@+=feaafiart1ev1aaatCvAUfKttLearuWrP9MDH5MBPbIqV92AaeXatLxBI9gBaebbnrfifHhDYfgasaacH8akY=wiFfYdH8Gipec8Eeeu0xXdbba9frFj0=OqFfea0dXdd9vqai=hGuQ8kuc9pgc9s8qqaq=dirpe0xb9q8qiLsFr0=vr0=vr0dc8meaabaqaciGacaGaaeqabaqabeGadaaakqaabeqaaiqbdMfazzaacaWaaSbaaSqaaiabd2eanjabdAeagjabdgeabjabikdaYaqabaGccqGGOaakcqWG0baDcqGGPaqkcqGH9aqpcqWGNbWzdaWgaaWcbaGaeeOta4KaeeizaqMaeeizaqMaeeymaedabeaakiabcIcaOiabdsha0jabcMcaPiabgUcaRiabdEgaNnaaBaaaleaacqqGnbqtcqqGJbWycqqGTbqBcqqGXaqmaeqaaOGaeiikaGIaemiDaqNaeiykaKIaey4kaSIaem4zaC2aaSbaaSqaaiabbofatjabbEha3jabbMgaPjabbwda1aqabaGccqGGOaakcqWG0baDcqGGPaqkcqGHRaWkcqWGNbWzdaWgaaWcbaGaeeOta4KaeeizaqMaeeizaqMaeeymaeJaeeilaWIaeeyta0Kaee4yamMaeeyBa0MaeeymaedabeaakiabcIcaOiabdsha0jabcMcaPiabgUcaRaqaaiaaxMaacaaMc8UaaGPaVlaaykW7caaMc8UaaGPaVlaaykW7cqWGNbWzdaWgaaWcbaGaeeOta4KaeeizaqMaeeizaqMaeeymaeJaeeilaWIaee4uamLaee4DaCNaeeyAaKMaeeynaudabeaakiabcIcaOiabdsha0jabcMcaPiabgUcaRiabdEgaNnaaBaaaleaacqqGnbqtcqqGJbWycqqGTbqBcqqGXaqmcqqGSaalcqqGtbWucqqG3bWDcqqGPbqAcqqG1aqnaeqaaOGaeiikaGIaemiDaqNaeiykaKIaey4kaSIaem4zaC2aaSbaaSqaaiabb6eaojabbsgaKjabbsgaKjabbgdaXiabbYcaSiabb2eanjabbogaJjabb2gaTjabbgdaXiabbYcaSiabbofatjabbEha3jabbMgaPjabbwda1aqabaGccqGGOaakcqWG0baDcqGGPaqkcqGHsislcqaH7oaBdaWgaaWcbaGaemyta0KaemOrayKaemyqaeKaeGOmaidabeaakiabdMfaznaaBaaaleaacqWGnbqtcqWGgbGrcqWGbbqqcqaIYaGmaeqaaOGaeiikaGIaemiDaqNaeiykaKIaey4kaSIaeqyTdu2aaSbaaSqaaiabd2eanjabdAeagjabdgeabjabikdaYaqabaGccqGGOaakcqWG0baDcqGGPaqkcqGGSaalcaWLjaGaaCzcamaabmaabaGaeGOmaidacaGLOaGaayzkaaaaaaa@C2AB@

where *ε*_*MFA*2_(*t*) denotes the noise (data uncertainty), *g*_Ndd1_(*t*), *g*_Mcm1_(*t*) and *g*_Swi5_(*t*) are the regulatory functions of transcription factors Ndd1, Mcm1, and Swi5 or the incident transcriptional regulations at the Ndd1, Mcm1, and Swi5 *cis *elements, respectively, *λ*_*MFA*2 _represents the mRNA decay rate of the target gene and we used the degradation rate measured by Wang *et al*. 2002 [20]. The *g*_Ndd1,Mcm1_(*t*), *g*_Ndd1,Swi5_(*t*), *g*_Mcm1,Swi5_(*t*) and *g*_Ndd1,Mcm1,Swi5_(*t*) denote the following nonlinear interactions among the three TFs:

*g*_Ndd1,Mcm1_(*t*) =: *f*(*g*_Ndd1_(*t*), *g*_Mcm1_(*t*)),

*g*_Ndd1,Swi5_(*t*) =: *f*(*g*_Ndd1_(*t*), *g*_Swi5_(*t*)),     (3)

*g*_Mcm1,Swi5_(*t*) =: *f*(*g*_Mcm1_(*t*), *g*_Swi5_(*t*)),

and

*g*_Ndd1,Mcm1,Swi5_(*t*) =: *f*(*g*_Ndd1_(*t*), *g*_Mcm1_(*t*), *g*_Swi5_(*t*)).     (4)

The biological meaning of Equation (2) is that the change in the mRNA expression level of gene *MFA2 *is due to the productions of regulatory functions of individual TFs and interactions among the TFs, i.e., *g*_Ndd1_(*t*) + *g*_Mcm1_(*t*) + *g*_Swi5_(*t*) + *g*_Ndd1,Mcm1_(*t*) + *g*_Ndd1,Swi5_(*t*) + *g*_Mcm1,Swi5_(*t*) + *g*_Ndd1,Mcm1,Swi5_(*t*), and -*λ*_*MFA*2_*Y*_*MFA*2_(*t*), which is the degradation of mRNA. Similarly, the *cis*-regulatory circuit of the target gene *CLB2 *in Figure 1b is modeled by

Y˙CLB2(t)=gFkh1(t)+gFkh2(t)+⋯+gFkh1,Fkh2(t)+⋯+gFhk1,Fkh2,Ndd1(t)+⋯     (5)                      −λCLB2YCLB2(t)+εCLB2(t).
 MathType@MTEF@5@5@+=feaafiart1ev1aaatCvAUfKttLearuWrP9MDH5MBPbIqV92AaeXatLxBI9gBaebbnrfifHhDYfgasaacH8akY=wiFfYdH8Gipec8Eeeu0xXdbba9frFj0=OqFfea0dXdd9vqai=hGuQ8kuc9pgc9s8qqaq=dirpe0xb9q8qiLsFr0=vr0=vr0dc8meaabaqaciGacaGaaeqabaqabeGadaaakqaabeqaaiqbdMfazzaacaWaaSbaaSqaaiabdoeadjabdYeamjabdkeacjabikdaYaqabaGccqGGOaakcqWG0baDcqGGPaqkcqGH9aqpcqWGNbWzdaWgaaWcbaGaeeOrayKaee4AaSMaeeiAaGMaeeymaedabeaakiabcIcaOiabdsha0jabcMcaPiabgUcaRiabdEgaNnaaBaaaleaacqqGgbGrcqqGRbWAcqqGObaAcqqGYaGmaeqaaOGaeiikaGIaemiDaqNaeiykaKIaey4kaSIaeS47IWKaey4kaSIaem4zaC2aaSbaaSqaaiabbAeagjabbUgaRjabbIgaOjabbgdaXiabbYcaSiabbAeagjabbUgaRjabbIgaOjabbkdaYaqabaGccqGGOaakcqWG0baDcqGGPaqkcqGHRaWkcqWIVlctcqGHRaWkcqWGNbWzdaWgaaWcbaGaeeOrayKaeeiAaGMaee4AaSMaeeymaeJaeeilaWIaeeOrayKaee4AaSMaeeiAaGMaeeOmaiJaeeilaWIaeeOta4KaeeizaqMaeeizaqMaeeymaedabeaakiabcIcaOiabdsha0jabcMcaPiabgUcaRiabl+UimjaaxMaacaWLjaWaaeWaaeaacqaI1aqnaiaawIcacaGLPaaaaeaacaaMc8UaaGPaVlaaykW7caaMc8UaaGPaVlaaykW7caaMc8UaaGPaVlaaykW7caaMc8UaaGPaVlaaykW7caaMc8UaaGPaVlaaykW7caaMc8UaaGPaVlaaykW7caaMc8UaaGPaVlaaykW7caaMc8UaeyOeI0Iaeq4UdW2aaSbaaSqaaiabdoeadjabdYeamjabdkeacjabikdaYaqabaGccqWGzbqwdaWgaaWcbaGaem4qamKaemitaWKaemOqaiKaeGOmaidabeaakiabcIcaOiabdsha0jabcMcaPiabgUcaRiabew7aLnaaBaaaleaacqWGdbWqcqWGmbatcqWGcbGqcqaIYaGmaeqaaOGaeiikaGIaemiDaqNaeiykaKIaeiOla4caaaa@B83F@

For simplicity, the indices of target genes and *cis *elements are denoted by numerical notation, so that the *cis*-regulatory circuit of gene *i *can be written as

Y˙i(t)=∑pvgp(t)+∑pqgp,q(t)+∑pqrgp,q,r(t)+⋯−λiYi(t)+εi(t),     (6)
 MathType@MTEF@5@5@+=feaafiart1ev1aaatCvAUfKttLearuWrP9MDH5MBPbIqV92AaeXatLxBI9gBaebbnrfifHhDYfgasaacH8akY=wiFfYdH8Gipec8Eeeu0xXdbba9frFj0=OqFfea0dXdd9vqai=hGuQ8kuc9pgc9s8qqaq=dirpe0xb9q8qiLsFr0=vr0=vr0dc8meaabaqaciGacaGaaeqabaqabeGadaaakeaacuWGzbqwgaGaamaaBaaaleaacqWGPbqAaeqaaOGaeiikaGIaemiDaqNaeiykaKIaeyypa0ZaaabCaeaacqWGNbWzdaWgaaWcbaGaemiCaahabeaaaeaacqWGWbaCaeaacqWG2bGDa0GaeyyeIuoakiabcIcaOiabdsha0jabcMcaPiabgUcaRmaaqahabaGaem4zaC2aaSbaaSqaaiabdchaWjabcYcaSiabdghaXbqabaaabaGaemiCaaNaemyCaehabaaaniabggHiLdGccqGGOaakcqWG0baDcqGGPaqkcqGHRaWkdaaeWbqaaiabdEgaNnaaBaaaleaacqWGWbaCcqGGSaalcqWGXbqCcqGGSaalcqWGYbGCaeqaaaqaaiabdchaWjabdghaXjabdkhaYbqaaaqdcqGHris5aOGaeiikaGIaemiDaqNaeiykaKIaey4kaSIaeS47IWKaeyOeI0Iaeq4UdW2aaSbaaSqaaiabdMgaPbqabaGccqWGzbqwdaWgaaWcbaGaemyAaKgabeaakiabcIcaOiabdsha0jabcMcaPiabgUcaRiabew7aLnaaBaaaleaacqWGPbqAaeqaaOGaeiikaGIaemiDaqNaeiykaKIaeiilaWIaaCzcaiaaxMaadaqadaqaaiabiAda2aGaayjkaiaawMcaaaaa@780B@

where *v *is the total number of *cis *elements in gene *i *and its corresponding degradation rate *λ*_*i *_measured by Wang *et al. *2002 [20]. If *λ*_*i *_is unavailable, it should be estimated together with the parameters *g*_*p*_(*t*), *g*_*p*,*q*_(*t*), *g*_*p*,*q*,*r*_(*t*) (see Methods). For the *cis*-regulatory circuits in Equations (2), (5) and (6), one obviously cannot estimate the multiple unknowns *g*_*p*_(*t*), *g*_*p*,*q*_(*t*), *g*_*p*,*q*,*r*_(*t*), … by only the expression profile (i.e., *Y*_*i*_(*t*)) of the *i*^th ^target gene. However, since the functions *g*_*p*_(*t*), *g*_*p*,*q*_(*t*), *g*_*p*,*q*,*r*_(*t*), … are assumed to be the same for all genes in the RGC and since there are overlaps of *cis *elements among genes in this RGC, one can estimate these functions from an array of expression profiles *Y*_1_(*t*), *Y*_2_(*t*), …, *Y*_*N*_(*t*) of the genes in the RGC simultaneously, taking advantage of cross information enhancement. The RGCs for *MFA2 *and *CLB2 *are shown in Table 1 and Table 2, respectively. In this situation, a cross-gene identification method is proposed as follows. By integrating the dynamic equations of *cis*-regulatory circuits for *N *genes in the RGC of the gene of interest, we obtain the following array of dynamic equations

(Y˙1(t)Y˙2(t)⋮⋮Y˙i(t)⋮⋮Y˙N(t))=(10⋯01⋯0⋯111⋯11⋯1⋯0⋮⋮⋮⋮⋮⋮⋮⋮01⋯00⋯0⋯1⋮⋮⋮⋮⋮⋮⋮⋮11⋯10⋯0⋯1)•(g1(t)g2(t)⋮g1,2(t)g1,3(t)⋮g1,2,3(t)⋮gp,q,r(t))−(λ1Y1(t)λ2Y2(t)⋮⋮λiYi(t)⋮⋮λNYN(t))+(ε1(t)ε2(t)⋮⋮εi(t)⋮⋮εN(t)).     (7)
 MathType@MTEF@5@5@+=feaafiart1ev1aaatCvAUfKttLearuWrP9MDH5MBPbIqV92AaeXatLxBI9gBaebbnrfifHhDYfgasaacH8akY=wiFfYdH8Gipec8Eeeu0xXdbba9frFj0=OqFfea0dXdd9vqai=hGuQ8kuc9pgc9s8qqaq=dirpe0xb9q8qiLsFr0=vr0=vr0dc8meaabaqaciaacaGaaeqabaqabeGadaaakeaadaqadaabaiqabaGafmywaKLbaiaadaWgaaWcbaGaeGymaedabeaakiabcIcaOiabdsha0jabcMcaPaqaaiqbdMfazzaacaWaaSbaaSqaaiabikdaYaqabaGccqGGOaakcqWG0baDcqGGPaqkaeaacqWIUlstaeaacqWIUlstaeaacuWGzbqwgaGaamaaBaaaleaacqWGPbqAaeqaaOGaeiikaGIaemiDaqNaeiykaKcabaGaeSO7I0eabaGaeSO7I0eabaGafmywaKLbaiaadaWgaaWcbaGaemOta4eabeaakiabcIcaOiabdsha0jabcMcaPaaacaGLOaGaayzkaaGaeyypa0ZaaeWaaeaafaqabeacjaaaaaaaaeaacqaIXaqmaeaacqaIWaamaeaacqWIVlctaeaacqaIWaamaeaacqaIXaqmaeaacqWIVlctaeaacqaIWaamaeaacqWIVlctaeaacqaIXaqmaeaacqaIXaqmaeaacqaIXaqmaeaacqWIVlctaeaacqaIXaqmaeaacqaIXaqmaeaacqWIVlctaeaacqaIXaqmaeaacqWIVlctaeaacqaIWaamaeaaaeaacqWIUlstaeaaaeaaaeaacqWIUlstaeaaaeaacqWIUlstaeaaaeaacqWIUlstaeaaaeaacqWIUlstaeaaaeaaaeaacqWIUlstaeaaaeaacqWIUlstaeaaaeaacqWIUlstaeaacqaIWaamaeaacqaIXaqmaeaacqWIVlctaeaacqaIWaamaeaacqaIWaamaeaacqWIVlctaeaacqaIWaamaeaacqWIVlctaeaacqaIXaqmaeaaaeaacqWIUlstaeaaaeaaaeaacqWIUlstaeaaaeaacqWIUlstaeaaaeaacqWIUlstaeaaaeaacqWIUlstaeaaaeaaaeaacqWIUlstaeaaaeaacqWIUlstaeaaaeaacqWIUlstaeaacqaIXaqmaeaacqaIXaqmaeaacqWIVlctaeaacqaIXaqmaeaacqaIWaamaeaacqWIVlctaeaacqaIWaamaeaacqWIVlctaeaacqaIXaqmaaaacaGLOaGaayzkaaGaeyOiGC7aaeWaaeaafaqabeGabaaaeaGabeaacqWGNbWzdaWgaaWcbaGaeGymaedabeaakiabcIcaOiabdsha0jabcMcaPaqaaiabdEgaNnaaBaaaleaacqaIYaGmaeqaaOGaeiikaGIaemiDaqNaeiykaKcabaGaeSO7I0eabaGaem4zaC2aaSbaaSqaaiabigdaXiabcYcaSiabikdaYaqabaGccqGGOaakcqWG0baDcqGGPaqkaeaacqWGNbWzdaWgaaWcbaGaeGymaeJaeiilaWIaeG4mamdabeaakiabcIcaOiabdsha0jabcMcaPaqaaiabl6UinbqaaiabdEgaNnaaBaaaleaacqaIXaqmcqGGSaalcqaIYaGmcqGGSaalcqaIZaWmaeqaaOGaeiikaGIaemiDaqNaeiykaKcabaGaeSO7I0eaaeaacqWGNbWzdaWgaaWcbaGaemiCaaNaeiilaWIaemyCaeNaeiilaWIaemOCaihabeaakiabcIcaOiabdsha0jabcMcaPaaaaiaawIcacaGLPaaacqGHsisldaqadaabaiqabaGaeq4UdW2aaSbaaSqaaiabigdaXaqabaGccqWGzbqwdaWgaaWcbaGaeGymaedabeaakiabcIcaOiabdsha0jabcMcaPaqaaiabeU7aSnaaBaaaleaacqaIYaGmaeqaaOGaemywaK1aaSbaaSqaaiabikdaYaqabaGccqGGOaakcqWG0baDcqGGPaqkaeaacqWIUlstaeaacqWIUlstaeaacqaH7oaBdaWgaaWcbaGaemyAaKgabeaakiabdMfaznaaBaaaleaacqWGPbqAaeqaaOGaeiikaGIaemiDaqNaeiykaKcabaGaeSO7I0eabaGaeSO7I0eabaGaeq4UdW2aaSbaaSqaaiabd6eaobqabaGccqWGzbqwdaWgaaWcbaGaemOta4eabeaakiabcIcaOiabdsha0jabcMcaPaaacaGLOaGaayzkaaGaey4kaSYaaeWaaqaaceqaaiabew7aLnaaBaaaleaacqaIXaqmaeqaaOGaeiikaGIaemiDaqNaeiykaKcabaGaeqyTdu2aaSbaaSqaaiabikdaYaqabaGccqGGOaakcqWG0baDcqGGPaqkaeaacqWIUlstaeaacqWIUlstaeaacqaH1oqzdaWgaaWcbaGaemyAaKgabeaakiabcIcaOiabdsha0jabcMcaPaqaaiabl6Uinbqaaiabl6Uinbqaaiabew7aLnaaBaaaleaacqWGobGtaeqaaOGaeiikaGIaemiDaqNaeiykaKcaaiaawIcacaGLPaaacqGGUaGlcaWLjaGaaCzcamaabmaabaGaeG4naCdacaGLOaGaayzkaaaaaa@277F@

In the cross-gene dynamic equations in (7), the process to identify regulatory functions *g*_*p*_(*t*), *g*_*p*,*q*_(*t*) and *g*_*p*,*q*,*r*_(*t*) from microarray data *Y*_*i*_(*t*), *i *= 1, 2,..., *N is *called the *cross-gene identification *approach, in which the regulatory functions *g*_*p*_(*t*), *g*_*p*,*q*_(*t*) and *g*_*p*,*q*,*r*_(*t*) are shared by genes in the RGC. Therefore, the estimation of the regulatory functions of one gene can also use the information from the profiles *Y*_1_(*t*), *Y*_2_(*t*), …, *Y*_*N*_(*t*) of other genes in RGC to improve the identification ability of the regulatory functions to reconstruct the *cis*-regulatory circuit of the gene of interest, which is called *cross information enhancement*.

**Remark 1 **: Suppose that the gene of interest in Equation (6) has *cis *elements *p *= 1,..., *v*. Then all genes whose *cis *elements are subsets of these *v cis *elements are included in the same RGC of the gene of interest.

### Cross-gene identification scheme

Since the number of functions *g*_*p*_(*t*), *g*_*p*,*q*_(*t*), *g*_*p*,*q*,*r*_(*t*), … is finite, we can estimate these functions if the number *N *of dynamic equations in Equation (7) is large enough. Equation (7) can be rewritten in an algebraic form

X(*t*) = A·G(*t*) + E(*t*),     (8)

where

X(t)=(Y˙1(t)+λ1Y1(t)Y˙2(t)+λ2Y2(t)⋮⋮Y˙i(t)+λiYi(t)⋮⋮Y˙N(t)+λNYN(t)),        G(t)=(g1(t)g2(t)⋮g1,2(t)g1,3(t)⋮g1,2,3(t)⋮gp,q,r(t)),        E(t)=(ε1(t)ε2(t)⋮⋮εi(t)⋮⋮εN(t))
 MathType@MTEF@5@5@+=feaafiart1ev1aaatCvAUfKttLearuWrP9MDH5MBPbIqV92AaeXatLxBI9gBaebbnrfifHhDYfgasaacH8akY=wiFfYdH8Gipec8Eeeu0xXdbba9frFj0=OqFfea0dXdd9vqai=hGuQ8kuc9pgc9s8qqaq=dirpe0xb9q8qiLsFr0=vr0=vr0dc8meaabaqaciGacaGaaeqabaqabeGadaaakeaacqqGybawcqGGOaakcqWG0baDcqGGPaqkcqGH9aqpdaqadaabaiqabaGafmywaKLbaiaadaWgaaWcbaGaeGymaedabeaakiabcIcaOiabdsha0jabcMcaPiabgUcaRiabeU7aSnaaBaaaleaacqaIXaqmaeqaaOGaemywaK1aaSbaaSqaaiabigdaXaqabaGccqGGOaakcqWG0baDcqGGPaqkaeaacuWGzbqwgaGaamaaBaaaleaacqaIYaGmaeqaaOGaeiikaGIaemiDaqNaeiykaKIaey4kaSIaeq4UdW2aaSbaaSqaaiabikdaYaqabaGccqWGzbqwdaWgaaWcbaGaeGOmaidabeaakiabcIcaOiabdsha0jabcMcaPaqaaiabl6Uinbqaaiabl6UinbqaaiqbdMfazzaacaWaaSbaaSqaaiabdMgaPbqabaGccqGGOaakcqWG0baDcqGGPaqkcqGHRaWkcqaH7oaBdaWgaaWcbaGaemyAaKgabeaakiabdMfaznaaBaaaleaacqWGPbqAaeqaaOGaeiikaGIaemiDaqNaeiykaKcabaGaeSO7I0eabaGaeSO7I0eabaGafmywaKLbaiaadaWgaaWcbaGaemOta4eabeaakiabcIcaOiabdsha0jabcMcaPiabgUcaRiabeU7aSnaaBaaaleaacqWGobGtaeqaaOGaemywaK1aaSbaaSqaaiabd6eaobqabaGccqGGOaakcqWG0baDcqGGPaqkaaGaayjkaiaawMcaaiabcYcaSiaaykW7caaMc8UaaGPaVlaaykW7caaMc8UaaGPaVlaaykW7caaMc8+exLMBbXgBcf2CPn2qVrwzqf2zLnharyGvLjhzH5wyaGabaiaa=DeacqGGOaakcqWG0baDcqGGPaqkcqGH9aqpdaqadaabaiqabaGaem4zaC2aaSbaaSqaaiabigdaXaqabaGccqGGOaakcqWG0baDcqGGPaqkaeaacqWGNbWzdaWgaaWcbaGaeGOmaidabeaakiabcIcaOiabdsha0jabcMcaPaqaaiabl6UinbqaaiabdEgaNnaaBaaaleaacqaIXaqmcqGGSaalcqaIYaGmaeqaaOGaeiikaGIaemiDaqNaeiykaKcabaGaem4zaC2aaSbaaSqaaiabigdaXiabcYcaSiabiodaZaqabaGccqGGOaakcqWG0baDcqGGPaqkaeaacqWIUlstaeaacqWGNbWzdaWgaaWcbaGaeGymaeJaeiilaWIaeGOmaiJaeiilaWIaeG4mamdabeaakiabcIcaOiabdsha0jabcMcaPaqaaiabl6UinbqaaiabdEgaNnaaBaaaleaacqWGWbaCcqGGSaalcqWGXbqCcqGGSaalcqWGYbGCaeqaaOGaeiikaGIaemiDaqNaeiykaKcaaiaawIcacaGLPaaacqGGSaalcaaMc8UaaGPaVlaaykW7caaMc8UaaGPaVlaaykW7caaMc8UaaGPaVlabbweafjabcIcaOiabdsha0jabcMcaPiabg2da9maabmaaeaGabeaacqaH1oqzdaWgaaWcbaGaeGymaedabeaakiabcIcaOiabdsha0jabcMcaPaqaaiabew7aLnaaBaaaleaacqaIYaGmaeqaaOGaeiikaGIaemiDaqNaeiykaKcabaGaeSO7I0eabaGaeSO7I0eabaGaeqyTdu2aaSbaaSqaaiabdMgaPbqabaGccqGGOaakcqWG0baDcqGGPaqkaeaacqWIUlstaeaacqWIUlstaeaacqaH1oqzdaWgaaWcbaGaemOta4eabeaakiabcIcaOiabdsha0jabcMcaPaaacaGLOaGaayzkaaaaaa@FECF@

**Remark 2 **: In order to calculate the derivatives in X(*t*) from undersampled data, a cubic spline interpolation method [21,22] is employed for curve fitting to obtain more accurate differential values and to learn more reliable models.

**Figure 2:**
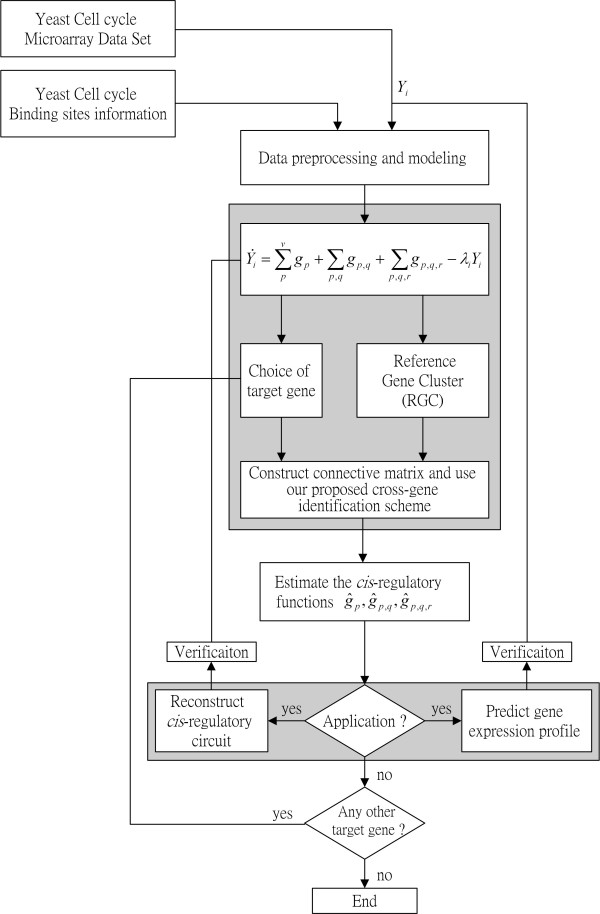
The overall flowchart of the modeling, identification and prediction of a *cis*-regulatory circuit.

In Equation (8), X(*t*) can be calculated from microarray data for the RGC of the gene of interest, which can then be used to estimate the vector G(*t*) by the least squares method, leading to the following solution:

G^(t)=(ATA)-1ATX(t)     (9)
 MathType@MTEF@5@5@+=feaafiart1ev1aaatCvAUfKttLearuWrP9MDH5MBPbIqV92AaeXatLxBI9gBaebbnrfifHhDYfgasaacH8akY=wiFfYdH8Gipec8Eeeu0xXdbba9frFj0=OqFfea0dXdd9vqai=hGuQ8kuc9pgc9s8qqaq=dirpe0xb9q8qiLsFr0=vr0=vr0dc8meaabaqaciGacaGaaeqabaqabeGadaaakeaacuqGhbWrgaqcaiabcIcaOiabdsha0jabcMcaPiabg2da9iabcIcaOiabbgeabnaaCaaaleqabaGaemivaqfaaOGaeeyqaeKaeeykaKYaaWbaaSqabeaacqqGTaqlcqqGXaqmaaGccqqGbbqqdaahaaWcbeqaaiabdsfaubaakiabbIfayjabcIcaOiabdsha0jabcMcaPiaaxMaacaWLjaWaaeWaaeaacqaI5aqoaiaawIcacaGLPaaaaaa@43C0@

for all *t*. After G^(t)
 MathType@MTEF@5@5@+=feaafiart1ev1aaatCvAUfKttLearuWrP9MDH5MBPbIqV92AaeXatLxBI9gBaebbnrfifHhDYfgasaacH8akY=wiFfYdH8Gipec8Eeeu0xXdbba9frFj0=OqFfea0dXdd9vqai=hGuQ8kuc9pgc9s8qqaq=dirpe0xb9q8qiLsFr0=vr0=vr0dc8meaabaqaciGacaGaaeqabaqabeGadaaakeaacuqGhbWrgaqcaiabcIcaOiabdsha0jabcMcaPaaa@30F6@ is estimated from Equation (9), the regulatory functions *g*_1_(*t*), …, *g*_1,2_(*t*), … and *g*_*p*,*q*,*r*_(*t*) in Equation (8) can be reconstructed for the genes in the RGC at every time point. If a *cis*-regulatory circuit is free of any function *g*_*p*_(*t*), *g*_*p*,*q*_(*t*), or *g*_*p*,*q*,*r*_(*t*), the value of the estimated function should be very small or zero. After the functions are estimated, they can be plugged into Equation (6) and the reconstruction of the *cis*-regulatory circuit of the gene of interest is completed. The flowchart for modeling, identifying and predicting a *cis*-regulatory circuit is shown in Figure 2.

In order to obtain more accurate *cis*-regulatory circuits, the model should include the triple interactions among the recognizing TFs; i.e., the vector G(*t*) in Equation (7) should include *g*_*p*,*q*,*r*_(*t*).

**Remark 3: **If the degradation parameters *λ*_*i *_in (6) are unavailable, the estimation procedure of G(*t*) and *λ*_*i *_from equation (7) to equation (9) should be modified as in Methods.

### Two application examples

#### I. The *cis*-regulatory circuit of *MFA2*

Suppose that the *cis*-regulatory circuit of the *MFA2 *gene is of interest. We construct a dynamic model of the *cis*-regulatory circuits of the genes in the RGC of *MFA2 *in Table 1 and then estimate the regulatory functions and interactions by the cross-gene identification scheme. The estimated transcriptional regulatory functions *g*_*p*_(*t*) and interactions *g*_*p*,*q*_(*t*) and *g*_*p*,*q*,*r*_(*t*) are shown as the insets in Figure 1a. These insets indicate that *cis *elements Ndd1, Mcm1 and Swi5 in *MFA2 *all have cell cycle regulatory abilities in the late G1 phase. In addition, every individual *cis *element has a positive regulatory function on the *MFA2 *gene; for example, the function *g*_4_(*t*) for Mcm1 has an obvious peak value in the transition phase late G1 of the cell cycle. Michaelis and Herskowitz [19] found that the *MFA2 *gene causes the cell cycle arrest at the G1 phase and is required for mating in yeast. Note that the interaction *g*_3,6_(*t*) between TFs Ndd1 and Swi5 is very weak or absent in the cell cycle. In contrast, the interaction *g*_4,6_(*t*) between TFs Mcm1 and Swi5 is dynamic; it has a high positive peak value in the late G1 phase, which coincides with *MFA2*'s activity phase. This interaction seems to play an important role of positive regulation in this *cis*-regulatory circuit. On the other hand, the regulatory function *g*_3,4,6_(*t*) of the interaction among TFs Ndd1, Mcm1 and Swi5 is negative on gene *MFA2*. This regulation may repress the expression of gene *MFA2 *to make the expression decay to the steady state. If there is no repression function such as *g*_3,4,6_(*t*), the expression of *MFA2 *will increase and may disrupt in the cell cycle.

#### II. The *cis*-regulatory circuit of *CLB2*

**Figure 3:**
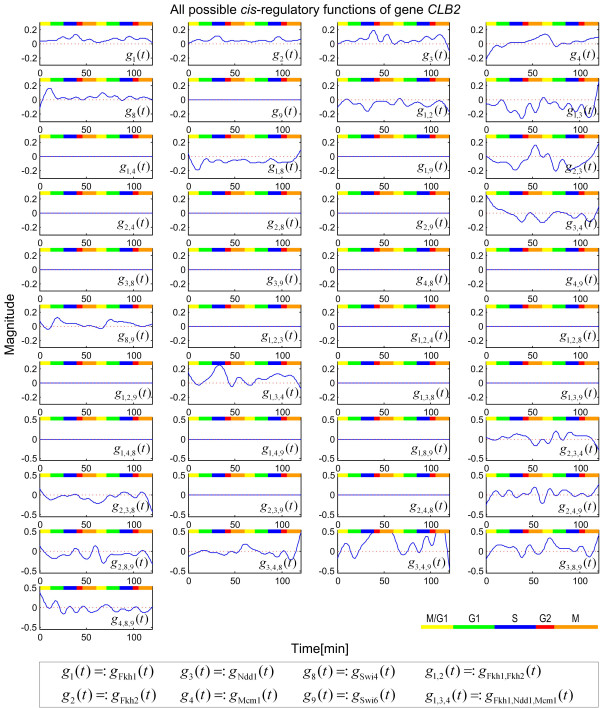
All estimated *cis*-regulatory functions, including the regulatory function *g*_*p*_(*t*) of each individual TF and the interactions *g*_*p*,*q*_(*t*) and *g*_*p*,*q*,*r*_(*t*) among the TFs that recognize the *cis *elements of the *CLB2 *gene. The numerical notation of regulatory functions is given in the box at the bottom of the figure. Different phases in the cell cycle are indicated by the colored bar near the right lower corner.

Clb proteins are crucial cyclins for completing the G2/M transition of the mitotic cell cycle and the most typical one is the B-type mitotic cyclin Clb2, which is required for entry into mitosis [23]. Suppose that the *cis*-regulatory circuit of gene *CLB2 *is of interest. Fkh1, Fkh2, Ndd1, Mcm1, Swi4 and Swi6 have been identified as the TFs that bind to the promoter sequence of *CLB2*[3,4,24,25]. As shown in Figure 1b, the possible *cis*-regulatory circuit of *CLB2 *is very complex. Using the cross-gene identification scheme, we reconstructed the *cis*-regulatory functions shown in Figure 3. Although there are 41 possible regulatory functions, including *g*_*p*_(*t*), *g*_*p*,*q*_(*t*) and *g*_*p*,*q*,*r*_(*t*), only 20 regulatory functions are found to have nonzero values (Figure 3).

The two *cis *elements Fkh1 and Fkh2 are found to have very similar regulatory functions *g*_1_(*t*) and *g*_2_(*t*), in agreement with the experimental evidence that the forkhead family members Fkh1 and Fkh2 of transcription factors have overlapping roles in the control of the G2/M transition [25,26]. The regulatory function *g*_2_(*t*) of Fkh2 has a distinct cell cycle regulatory ability (Figure 3) and especially, the interaction function *g*_2,3_(*t*) between Fkh2 and Ndd1 has a strong regulatory contribution to the gene expression profile in the M/G1 phase. The regulatory function *g*_4_(*t*) of Mcm1 has two peaks. There is experimental evidence that Mcm1 is a member of an evolutionarily conserved class of transcription factors that have related to DNA binding sequences and dimerization domains. In addition, Mcm1 binds the early cell cycle box (ECB) that contains a Mcm1 *cis *element in the *SWI4*, *CLN3*, *CDC6*, and *CDC47 *promoters and activates M/G1-specific transcription [27].

The cell cycle genes that are activated during the late G1 or S phase have SBF or MBF sequence-specific transcription factors that bind the *cis *elements in their promoter region. SBF (the Swi4-Swi6 cell cycle box binding factor) is a heterodimer of Swi4 and Swi6 [3,28,29]. The regulatory functions *g*_8_(*t*) and *g*_9_(*t*) of Swi4 and Swi6 and their interaction function *g*_8,9_(*t*) are estimated using the dynamic expression model (Figure 3). It is well-known that neither Swi4 nor Swi6 alone has obvious cell cycle regulation ability, and indeed we found that *g*_8_(*t*) has only one peak and so shows no cycle and that *g*_9_(*t*) shows no capability of cell cycle regulation (Figure 3). In contrast, the combination of Swi6 and Swi4 to make the complex SBF enables the *cis *elements Swi6 and Swi4 to provide cell cycle regulation capacity; that is, the interaction function *g*_8,9_(*t*) of Swi6 and Swi4 has a peak during the G1/S phase of the cell cycle. Ndd1 and Fkh2 are bound to identical promoters throughout the cell cycle and their interaction *g*_2,3_(*t*) is an important transcriptional process targeted by the Cdk activity [24,30]. In addition, there is another obvious positive interaction *g*_3,4,9_(*t*) among Ndd1, Mcm1, and Swi6 (Figure 3). It has a large regulatory ability in the G1/S phase, which almost dominates the expression profile of *CLB2*. In contrast, *g*_3,4_(*t*) has a negative regulation contribution. We therefore propose that the key factor Swi6 in the interaction *g*_3,4,9_(*t*) is similar to its role in SBF and MBF. At any rate, our model suggests that Swi6 plays a key role in the interaction among Ndd1, Mcm1, and Swi6. This is a new finding in the *cis*-regulatory circuit of *CLB2*.

We also confirm the well-known interaction among Ndd1, Fkh2, and Mcm1 in the *cis*-regulatory circuits of the *CLB2 *and *SWI5 *genes because the interaction function *g*_2,3,4_(*t*) has a distinct regulatory ability in the G2/M phase of the cell cycle (Figure 3). Interestingly, the interaction function *g*_3,4,9_(*t*) among Ndd1, Mcm1 and Swi6 is about two times higher than any of the other functions in Figure 3. In addition, the interaction *g*_1,3,4_(*t*) among Fkh1, Ndd1 and Mcm1 is highly positive, the interaction *g*_3,4,8_(*t*) among Ndd1, Mcm1 and Swi4 is mildly positive, while the interaction *g*_3,8,9_(*t*) among Ndd1, Swi4 and Swi6 is negative. These observations are in agreement with the fact that the regulatory ability of an interaction among TFs is usually much stronger than that of an individual TF; in other words, there is synergy among TFs.

In summary, there are many experimental observations that support the *cis*-regulatory functions identified by the dynamic model and our model provides novel insights into the quantitative regulation of the *cis*-regulatory circuit of a gene of interest.

### Support from expression phases of TFs in the cell cycle

**Figure 4:**
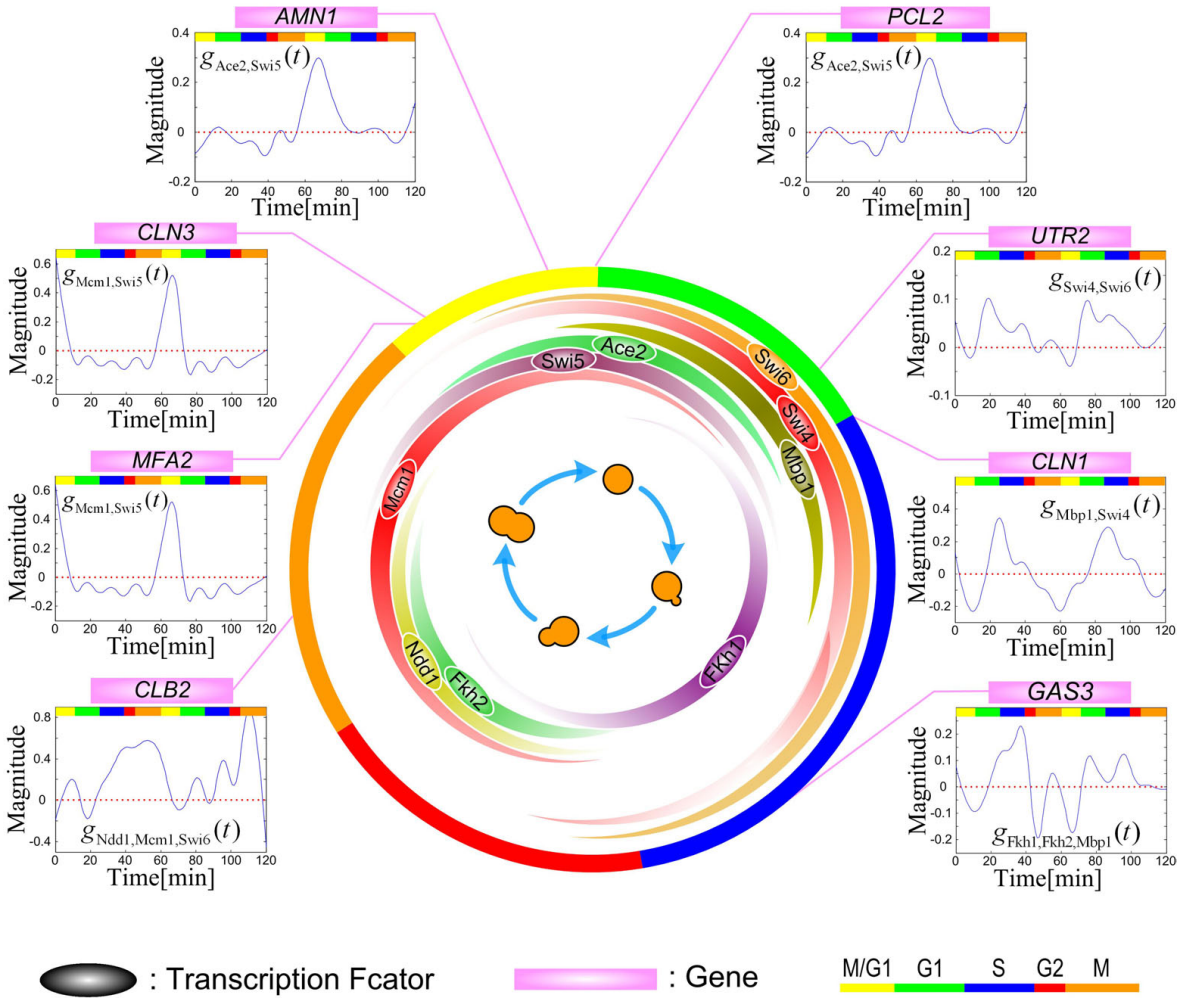
The gene expression phases match the main regulatory functions of TFs. It is seen that the main interaction functions of TFs have a peak value and always occur during or soon after the mRNA expression phases of the corresponding genes. For example, the gene *MFA2 *has the main interaction regulatory function *g*_Mcm1,Swi5_(*t*) which has a peak during the expression phases between the TFs Mcm1 and Swi5 by identifying the *cis*-regulatory circuit. As another example, the gene *UTR2 *has the main interaction regulatory function *g*_*Swi*4,*Swi*6_(*t*), which has a peak during the expression phases between the TFs Swi4 and Swi6 by identifying the *cis*-regulatory circuit. These results indicate that the main regulatory functions have a peak value phase to match the gene expression phase. Therefore, we can estimate the gene expression phase by identifying the main regulatory function. The width of a colored band in the inner circle is approximately proportional to the expression level of the TF gene of interest in the cell cycle. A pink line points to the main expression phase of a target gene in the pink box. Different phases in the cell cycle are indicated by the colored bar at the right lower corner.

In this paper, the question of why the strengths of regulatory functions in the *cis*-regulatory circuits are different in different phases of the cell cycle is investigated. Based on the mRNA expression profiles of transcription factor genes from experiments, the distribution of the expressions of the nine TF genes in the different phases of the cell cycle is shown in Figure 4. In support of our results, the large positive interaction functions (peaks) among a set of TFs always occur during the expression phases of the genes of the interacting TFs. For example, for the *cis*-regulatory circuit of *MFA2 *(Figure 1a), there is an obvious peak for the function *g*_4,6_(*t*) of the interaction between TFs Mcm1 and Swi5 during the M phase, and in Figure 4, this peak indeed occurs during the expression phases of the two TF genes [18,27]. As another example, for the *cis*-regulatory circuit of *CLB2*, there is a strong interaction (*g*_3,4,9 _(*t*)) among Ndd1, Mcm1 and Swi6 starting from the G2 phase (Figure 3). We can therefore infer that the expression of a cell cycle gene in a specific phase of the cell cycle needs a specific inducing signal, which is mainly from the interactions of certain specific TFs that bind the *cis *elements of the gene.

### Accuracy of reconstructed *cis*-regulatory circuits

The accuracy of the reconstructed *cis*-regulatory circuit of a gene can be evaluated by reconstructing the expression profile of the gene using the reconstructed *cis*-regulatory circuit

**Figure 5:**
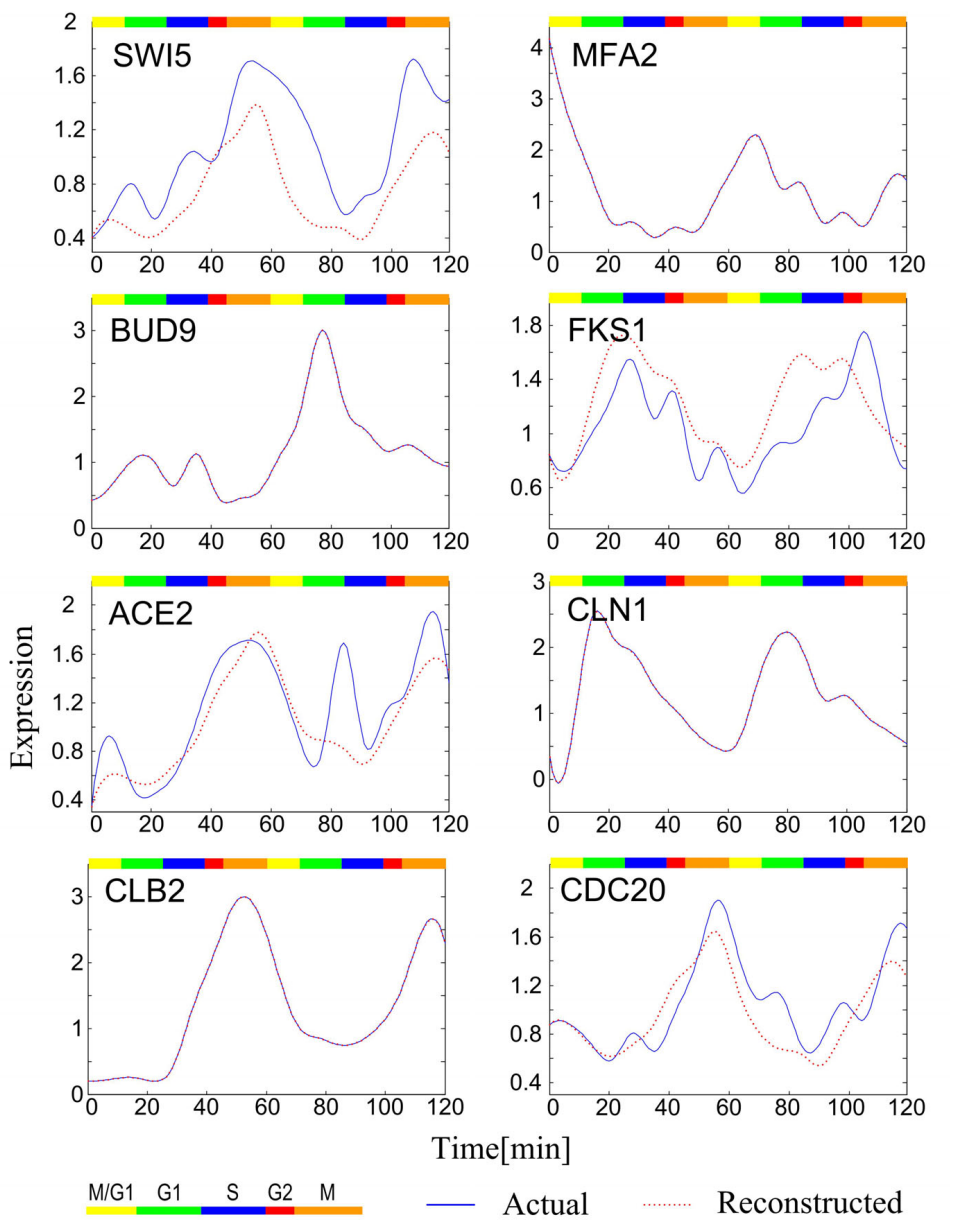
Comparison between the actual gene expression profiles (with cubic spline) and the reconstructed gene expression profiles. The examples shown were randomly chosen. The reconstructed gene expression profiles were obtained by integrating the estimated *cis*-regulatory functions and the chromatin immunoprecipitation data. When there is only one blue line in a figure it means that the reconstructed function is very close to the actual gene expression profile. Different phases in the cell cycle are indicated by the colored bar.

Y^˙i(t)=∑pvg^p(t)+∑pqg^p,q(t)+∑pqrg^p,q,r(t)+⋯−λiY^i(t),     (10)
 MathType@MTEF@5@5@+=feaafiart1ev1aaatCvAUfKttLearuWrP9MDH5MBPbIqV92AaeXatLxBI9gBaebbnrfifHhDYfgasaacH8akY=wiFfYdH8Gipec8Eeeu0xXdbba9frFj0=OqFfea0dXdd9vqai=hGuQ8kuc9pgc9s8qqaq=dirpe0xb9q8qiLsFr0=vr0=vr0dc8meaabaqaciGacaGaaeqabaqabeGadaaakeaacuWGzbqwgaqcgaGaamaaBaaaleaacqWGPbqAaeqaaOGaeiikaGIaemiDaqNaeiykaKIaeyypa0ZaaabCaeaacuWGNbWzgaqcamaaBaaaleaacqWGWbaCaeqaaaqaaiabdchaWbqaaiabdAha2bqdcqGHris5aOGaeiikaGIaemiDaqNaeiykaKIaey4kaSYaaabCaeaacuWGNbWzgaqcamaaBaaaleaacqWGWbaCcqGGSaalcqWGXbqCaeqaaOGaeiikaGIaemiDaqNaeiykaKcaleaacqWGWbaCcqWGXbqCaeaaa0GaeyyeIuoakiabgUcaRmaaqahabaGafm4zaCMbaKaadaWgaaWcbaGaemiCaaNaeiilaWIaemyCaeNaeiilaWIaemOCaihabeaaaeaacqWGWbaCcqWGXbqCcqWGYbGCaeaaa0GaeyyeIuoakiabcIcaOiabdsha0jabcMcaPiabgUcaRiabl+UimjabgkHiTiabeU7aSnaaBaaaleaacqWGPbqAaeqaaOGafmywaKLbaKaadaWgaaWcbaGaemyAaKgabeaakiabcIcaOiabdsha0jabcMcaPiabcYcaSiaaxMaacaWLjaWaaeWaaeaacqaIXaqmcqaIWaamaiaawIcacaGLPaaaaaa@7216@

where g^p(t)
 MathType@MTEF@5@5@+=feaafiart1ev1aaatCvAUfKttLearuWrP9MDH5MBPbIqV92AaeXatLxBI9gBaebbnrfifHhDYfgasaacH8akY=wiFfYdH8Gipec8Eeeu0xXdbba9frFj0=OqFfea0dXdd9vqai=hGuQ8kuc9pgc9s8qqaq=dirpe0xb9q8qiLsFr0=vr0=vr0dc8meaabaqaciGacaGaaeqabaqabeGadaaakeaacuWGNbWzgaqcamaaBaaaleaacqWGWbaCaeqaaOGaeiikaGIaemiDaqNaeiykaKcaaa@32D7@, g^p,q(t)
 MathType@MTEF@5@5@+=feaafiart1ev1aaatCvAUfKttLearuWrP9MDH5MBPbIqV92AaeXatLxBI9gBaebbnrfifHhDYfgasaacH8akY=wiFfYdH8Gipec8Eeeu0xXdbba9frFj0=OqFfea0dXdd9vqai=hGuQ8kuc9pgc9s8qqaq=dirpe0xb9q8qiLsFr0=vr0=vr0dc8meaabaqaciGacaGaaeqabaqabeGadaaakeaacuWGNbWzgaqcamaaBaaaleaacqWGWbaCcqGGSaalcqWGXbqCaeqaaOGaeiikaGIaemiDaqNaeiykaKcaaa@3522@ and g^p,q,r(t)
 MathType@MTEF@5@5@+=feaafiart1ev1aaatCvAUfKttLearuWrP9MDH5MBPbIqV92AaeXatLxBI9gBaebbnrfifHhDYfgasaacH8akY=wiFfYdH8Gipec8Eeeu0xXdbba9frFj0=OqFfea0dXdd9vqai=hGuQ8kuc9pgc9s8qqaq=dirpe0xb9q8qiLsFr0=vr0=vr0dc8meaabaqaciGacaGaaeqabaqabeGadaaakeaacuWGNbWzgaqcamaaBaaaleaacqWGWbaCcqGGSaalcqWGXbqCcqGGSaalcqWGYbGCaeqaaOGaeiikaGIaemiDaqNaeiykaKcaaa@376F@ have been estimated by the cross-gene identification scheme. The reconstructed profile and the observed profile are compared in Figure 5. We find that if the number of the *cis *elements of a gene is large enough, the reconstructed expression profile is very accurate; otherwise, it may be inaccurate. Fortunately, although the reconstructed expression profile is not accurate in some genes, the trend of the expression profile for a gene is always correct.

### Prediction of gene expression profile

In the above, each regulatory circuit was identified using 95% of genes in its RGC, and the remaining 5% of genes in the RGC will now be used for predicting expression profiles, i.e., for cross validation. Our cross-gene identification scheme assumes that the regulatory functions of TFs are universal in the cluster of genes with similar functions. Under this assumption, our method should be able to predict the expression profiles of other genes in the same cluster that have not been employed in Equation (7) to reconstruct the *cis*-regulatory circuits. This is one way to validate our model.

**Figure 6:**
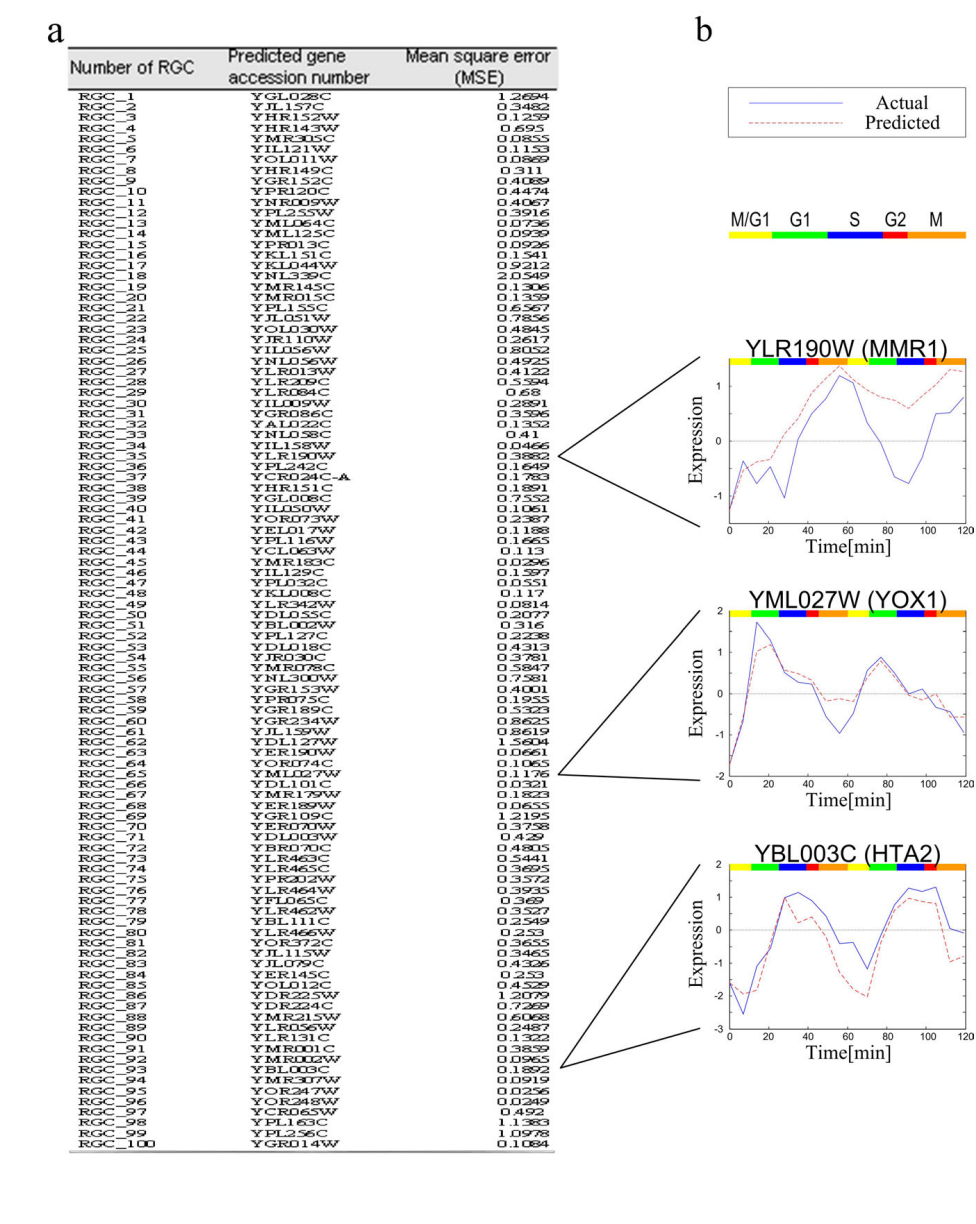
The regulatory functions integrated with ChIP-chip data to predict gene expression profiles. To test the prediction performance of our model, (a) 100 yeast cell cycle genes that have not been employed in the reconstruction of the *cis*-regulatory circuits are randomly chosen from their corresponding reference gene clusters (RGCs). The maximum mean square error (MSE) of prediction results is 2.055 and the minimum is 0.025. (b) Three examples of the comparison between the actual (blue) and the predicted (red) gene expression profiles. Different phases in the cell cycle are indicated by the colored bar.

In Figure 6, we randomly chose 100 RGCs to test the prediction accuracy; that is, for each RGC we show the prediction result for one of the unused genes. Figure 6a shows the predicted target gene and the mean square error (MSE) of prediction results which has the maximum of 2.055 and the minimum of 0.025. In addition, three examples of the detailed comparison between the actual and the predicted gene expression profiles are shown in Figure 6b. In general, the predicted profiles are satisfactory approximations of the observed expression profiles. We found that the smaller the number of the *cis *elements, the less accurate the prediction results. However, if some *cis *elements of a gene have strong regulatory functions, the expression profiles of this gene can be predicted accurately even when the number of *cis *elements is small. If some genes in the RGC have the same *cis *elements but have different observed gene expression profiles, these expression profiles will lead to poor estimation of parameters. This is the main cause of prediction error. Why does this situation arise? It may be that some *cis *elements of these genes have not yet been identified or there are some errors in the inference of the *cis *elements. For example, *MMR1 *may have another *cis *element Gcr2 [5] and this may be why the predicted profile is quite different from the observed profile (Figure 6b).

## Discussion

In contrast to current methods, our method uses all possible expression profile information from the cluster of genes to reconstruct the *cis*-regulatory circuit of a target gene. In particular, our method is capable of extracting dynamic interactions among TFs. For this reason, the analysis and interpretation of output expression profiles become straightforward. Therefore, our method has a high potential for applications such as studying variations of the *cis*-regulatory circuit of the same gene in different yeast strains to investigate the regulatory evolution of the gene.

The contributions of this study include: (1) a nonlinear dynamical model is developed for *cis*-regulatory circuits in terms of regulatory functions and interactions among TFs, (2) a cross-gene identification scheme is proposed to estimate many parameters involved in the dynamical model of *cis*-regulatory circuits from the expression profiles of genes in the reference gene cluster, (3) a detailed identification of the dynamic *cis*-regulatory abilities of TFs, which vary with time, and (4) a gene expression prediction method is developed by the proposed dynamic *cis*-regulatory circuit, assuming that the *cis*-regulatory functions of the same TFs in different circuits are the same. Three advantages of our method over current methods are that the *cis*-regulatory circuit is constructed with the circuit structure intact, that it uses the expression profiles of many genes simultaneously to obtain extra information, and that it is dynamic and quantitative.

Significantly, our model not only can confirm known regulations but also can provide conjectures for experimental verification. Consider the key positive *cis*-regulatory function *g*_4,6_(*t*) in Figure 1a. We propose that during the expression of gene *MFA2*, the transcription factor Ndd1 (the G2/M phase) communicates with the transcription factor Mcm1 (the M phase) to transmit a specific signal to induce the expression of the *MFA2 *gene. Such conjectures from the reconstructed *cis*-regulatory circuits may be useful for studying the regulatory evolution of genes by comparing the *cis*-regulatory functions of different strains, or for predicting the gene expression behavior before conducting an experiment.

However, we found poor results in some cases. For example, in Figure 3, we were unable to find the basal regulatory function *g*_9_(*t*) of individual Swi6 or the interaction *g*_2,4_(*t*) between Fkh2 and Mcm1 for gene *CLB2 *[31]. These regulatory functions have not been identified by our scheme because they have no obvious specific phase regulatory ability. Besides, from the *cis*-regulatory functions in Figures 1 and 2, several *cis*-regulatory function profiles did not show a clear periodicity. A possible reason may be that the original microarray data were noisy and the use of cubic spline interpolation and linear transformation of microarray data in our scheme had introduced new noise and distortions. Most probably, the *cis *element information used to construct the *cis*-regulatory circuits under yeast cell cycle is not complete; only nine significant *cis *elements were considered in this study to reduce the complexity of the mathematical model. Another possible source of error is that we have not considered the order of the *cis *elements on the promoter region, which may affect the strength of the interaction between TFs [36]. Such differences, however, can be incorporated by putting, say both *g*_*p*,*q*_(*t*) and *g*_*p*,*q*,*r*_(*t*), into the model.

In view of the facts that there are uncertainties about the *cis *elements of some of the genes studied and that microarray data are noisy, it is remarkable that our method gave accurate results for the expression profiles of the majority of the cell cycle genes studied and also gave fairly accurate predictions of the expression profiles of other cell cycle genes. In the future, if better *cis *elements data and more accurate and longer gene expression profile data become available, we should be able to improve the reconstruction of *cis*-regulatory circuits. Also, our approach may be extended to reconstruct *cis*-regulatory circuits in more diverse conditions and more complex eukaryotes. After *cis*-regulatory circuits are accurately described by explicit dynamical equations, some applications will be straightforward.

## Conclusion

In this study, we assume that the regulatory functions of the same *cis *elements and the interaction functions among their TFs are similar across genes within the cluster of genes with overlapping *cis *elements; i.e., the regulatory functions and interaction functions are universal in this cluster of genes. Under this assumption, the cross-gene identification scheme takes advantage of cross-information enhancement to improve the accuracy of parameter estimation. The number of genes used should be large enough, so that we have a large number of outputs (i.e., their microarray data) for parameter estimation.

After the parameters of the *cis*-regulatory circuits of interest have been estimated, the circuits can be explicitly described by plugging these parameters into their corresponding dynamic equations. Moreover, these estimated functions and interactions can be used to predict the expression profiles of other genes that share the same *cis*-regulatory elements but have not been used to identify the *cis*-regulatory circuits. In this manner, we can evaluate the performance of the proposed dynamic model of *cis*-regulatory circuits. From a number of examples, we have found that the predicted results are in most cases satisfactory, confirming the validity of the proposed dynamic model of *cis*-regulatory circuits. Our modeling represents a new approach to studying *cis*-regulatory circuits from cross-gene expression data. It is a systems biology approach because we consider the regulatory circuits of many genes and many TFs at the same time and we use system identification techniques to estimate the parameters of the circuits. The results of expression prediction from experimental data suggest that our novel approach is suitable for deciphering the regulatory functions and the cooperativity of the TFs that regulate the expression of a gene.

## Methods

### Experimental data

To identify the *cis*-regulatory circuit of a gene of interest in the yeast cell cycle, we apply our approach to the data of Spellman *et al*. 1998 [18], which contains expression profiles of 6178 open reading frames (ORFs) in the yeast *Saccharomyces cerevisiae *during the cell cycle [33]. Our analysis was applied to the *α*-factor arrest data set. The raw data were transformed into a linear scale from the original log ratio carried out by Spellman *et al*. 1998 [18]. To reduce the effect of noise and to overcome undersampled microarray data in the estimation of *cis*-regulatory circuits, the cubic spline was used for data interpolation and smoothing to obtain a less sensitive first derivative of the expression pattern and to learn a more reliable model. Furthermore, the noise is modeled in the noise term *ε*_*i*_(t) in Equation (7).

From the RGC of the gene of interest, the cross-gene identification method from Equations (8) to (9) is employed to reconstruct the *cis*-regulatory circuit. The connectivity information between TFs and their target genes was obtained from the yeast cell cycle analysis [4]. We focused on the nine transcription factors that have been identified to play important roles in the transcription regulation of a set of yeast genes whose expressions are cell-cycle dependent; these nine transcription factors are Mbp1, Swi4, Swi6, Mcm1, Fkh1, Fkh2, Ndd1, Swi5, and Ace2 [24,26,27,34] (See additional file 1: Table for the original data used to perform this analysis).

### Estimation of degradation rate

If the mRNA degradation rate *λ*_*i *_in Equation (6) has not been estimated experimentally, it should be estimated together with the parameters *g*_*p*_(*t*), *g*_*p*,*q*_(*t*), *g*_*p*,*q*,*r*_(*t*),. The algorithm to estimate the *λ*_*i *_is described as follows. First, Equation (6) is changed to

Y˙i(t)=∑pvgp(t)+∑pqgp,q(t)+∑pqrgp,q,r(t)+⋯−λi(t)Yi(t)+εi(t),     (11)
 MathType@MTEF@5@5@+=feaafiart1ev1aaatCvAUfKttLearuWrP9MDH5MBPbIqV92AaeXatLxBI9gBaebbnrfifHhDYfgasaacH8akY=wiFfYdH8Gipec8Eeeu0xXdbba9frFj0=OqFfea0dXdd9vqai=hGuQ8kuc9pgc9s8qqaq=dirpe0xb9q8qiLsFr0=vr0=vr0dc8meaabaqaciGacaGaaeqabaqabeGadaaakeaacuWGzbqwgaGaamaaBaaaleaacqWGPbqAaeqaaOGaeiikaGIaemiDaqNaeiykaKIaeyypa0ZaaabCaeaacqWGNbWzdaWgaaWcbaGaemiCaahabeaaaeaacqWGWbaCaeaacqWG2bGDa0GaeyyeIuoakiabcIcaOiabdsha0jabcMcaPiabgUcaRmaaqafabaGaem4zaC2aaSbaaSqaaiabdchaWjabcYcaSiabdghaXbqabaGccqGGOaakcqWG0baDcqGGPaqkaSqaaiabdchaWjabdghaXbqab0GaeyyeIuoakiabgUcaRmaaqafabaGaem4zaC2aaSbaaSqaaiabdchaWjabcYcaSiabdghaXjabcYcaSiabdkhaYbqabaaabaGaemiCaaNaemyCaeNaemOCaihabeqdcqGHris5aOGaeiikaGIaemiDaqNaeiykaKIaey4kaSIaeS47IWKaeyOeI0Iaeq4UdW2aaSbaaSqaaiabdMgaPbqabaGccqGGOaakcqWG0baDcqGGPaqkcqWGzbqwdaWgaaWcbaGaemyAaKgabeaakiabcIcaOiabdsha0jabcMcaPiabgUcaRiabew7aLnaaBaaaleaacqWGPbqAaeqaaOGaeiikaGIaemiDaqNaeiykaKIaeiilaWIaaCzcaiaaxMaadaqadaqaaiabigdaXiabigdaXaGaayjkaiaawMcaaaaa@7BEB@

where *v *is the total number of *cis *elements in gene *i*, and the degradation rate is substituted as *λ*_*i*_(*t*). Similarly, since the functions *g*_*p*_(*t*), *g*_*p*,*q*_(*t*), *g*_*p*,*q*,*r*_(*t*), … are assumed to be the same for all genes in the RGC and since there are overlaps of *cis *elements among genes in this RGC, one can estimate these functions from an array of expression profiles *Y*_1_(*t*), *Y*_2_(*t*), …, *Y*_*N*_(*t*) of the genes in the RGC simultaneously, taking advantage of cross information enhancement. The RGCs for *MFA2 *and *CLB2 *are shown in Table 1 and Table 2, respectively.

Second, by integrating the dynamic equations of *cis*-regulatory circuits for *N *genes in the RGC of the gene of interest, we obtain the following array of dynamic equations

(Y˙1(t)Y˙2(t)⋮⋮Y˙1(t)⋮⋮Y˙N(t))=(10⋯01⋯0⋯111⋯11⋯1⋯0⋮⋮⋮⋮⋮⋮01⋯00⋯0⋯1⋮⋮⋮⋮⋮⋮11⋯10⋯01|−Y1(t)0↔00−Y2(t)⋱↕−Y1(t)↕⋱00↔0−YN(t))•(g1(t)g2(t)⋮g1,2(t)g1,3(t)⋮g1,2,3(t)⋮gp,q,r(t)λ1(t)λ2(t)⋮⋮λi(t)⋮⋮λN(t))+(ε1(t)ε2(t)⋮⋮εi(t)⋮⋮εN(t)).     (12)
 MathType@MTEF@5@5@+=feaafiart1ev1aaatCvAUfKttLearuWrP9MDH5MBPbIqV92AaeXatLxBI9gBaebbnrfifHhDYfgasaacH8akY=wiFfYdH8Gipec8Eeeu0xXdbba9frFj0=OqFfea0dXdd9vqai=hGuQ8kuc9pgc9s8qqaq=dirpe0xb9q8qiLsFr0=vr0=vr0dc8meaabaqaciaacaGaaeqabaqabeGadaaakeaadaqadaqaauaabeqaieaaaaaabaGafmywaKLbaiaadaWgaaWcbaGaeGymaedabeaakiabcIcaOiabdsha0jabcMcaPaqaaiqbdMfazzaacaWaaSbaaSqaaiabikdaYaqabaGccqGGOaakcqWG0baDcqGGPaqkaeaacqWIUlstaeaacqWIUlstaeaacuWGzbqwgaGaamaaBaaaleaacqaIXaqmaeqaaOGaeiikaGIaemiDaqNaeiykaKcabaGaeSO7I0eabaGaeSO7I0eabaGafmywaKLbaiaadaWgaaWcbaGaemOta4eabeaakiabcIcaOiabdsha0jabcMcaPaaaaiaawIcacaGLPaaacqGH9aqpdaqadaqaamaaeiaabaqbaeqabGqcaaaaaaaabaGaeGymaedabaGaeGimaadabaGaeS47IWeabaGaeGimaadabaGaeGymaedabaGaeS47IWeabaGaeGimaadabaGaeS47IWeabaGaeGymaedabaGaeGymaedabaGaeGymaedabaGaeS47IWeabaGaeGymaedabaGaeGymaedabaGaeS47IWeabaGaeGymaedabaGaeS47IWeabaGaeGimaadabaaabaGaeSO7I0eabaaabaaabaGaeSO7I0eabaaabaaabaaabaGaeSO7I0eabaaabaGaeSO7I0eabaaabaaabaGaeSO7I0eabaaabaaabaaabaGaeSO7I0eabaGaeGimaadabaGaeGymaedabaGaeS47IWeabaGaeGimaadabaGaeGimaadabaGaeS47IWeabaGaeGimaadabaGaeS47IWeabaGaeGymaedabaaabaGaeSO7I0eabaaabaaabaGaeSO7I0eabaaabaaabaaabaGaeSO7I0eabaaabaGaeSO7I0eabaaabaaabaGaeSO7I0eabaaabaaabaaabaGaeSO7I0eabaGaeGymaedabaGaeGymaedabaGaeS47IWeabaGaeGymaedabaGaeGimaadabaGaeS47IWeabaGaeGimaadabaaabaGaeGymaedaaaGaayjcSdqbaeqabGGbaaaaaaqaaiabgkHiTiabdMfaznaaBaaaleaacqaIXaqmaeqaaOGaeiikaGIaemiDaqNaeiykaKcabaGaeGimaadabaaabaGaeyiLHSkabaaabaGaeGimaadabaGaeGimaadabaGaeyOeI0IaemywaK1aaSbaaSqaaiabikdaYaqabaGccqGGOaakcqWG0baDcqGGPaqkaeaaaeaaaeaaaeaaaeaaaeaaaeaacqWIXlYtaeaaaeaaaeaaaeaaaeaaaeaaaeaaaeaaaeaaaeaacqWIvgIyaeaaaeaaaeaacqGHsislcqWGzbqwdaWgaaWcbaGaeGymaedabeaakiabcIcaOiabdsha0jabcMcaPaqaaaqaaiablwziIbqaaaqaaaqaaaqaaaqaaiablgVipbqaaaqaaaqaaaqaaaqaaaqaaaqaaiabicdaWaqaaiabicdaWaqaaaqaaiabgsziRcqaaaqaaiabicdaWaqaaiabgkHiTiabdMfaznaaBaaaleaacqWGobGtaeqaaOGaeiikaGIaemiDaqNaeiykaKcaaaGaayjkaiaawMcaaiabgkci3oacaciaaamcbmaaeGaGacaaaJGabeacaciaaamccWaGacaaaJWGNbWzdGaGacaaaJWgaaWcbGaGacaaaJGamaiGaaaWiGymaedabKaGacaaaJaakiadaciaaamccIcaOiadaciaaamcdsha0jadaciaaamccMcaPaqaiaiGaaaWiiadaciaaamcdEgaNnacaciaaamcBaaaleacaciaaamccWaGacaaaJaIYaGmaeqcaciaaamcaOGamaiGaaaWiiikaGIamaiGaaaWimiDaqNamaiGaaaWiiykaKcabGaGacaaaJGamaiGaaaWiSO7I0eabGaGacaaaJGamaiGaaaWim4zaC2aiaiGaaaWiSbaaSqaiaiGaaaWiiadaciaaamcigdaXiadaciaaamccYcaSiadaciaaamcikdaYaqajaiGaaaWiaGccWaGacaaaJGGOaakcWaGacaaaJWG0baDcWaGacaaaJGGPaqkaeacaciaaamccWaGacaaaJWGNbWzdGaGacaaaJWgaaWcbGaGacaaaJGamaiGaaaWiGymaeJamaiGaaaWiiilaWIamaiGaaaWiG4mamdabKaGacaaaJaakiadaciaaamccIcaOiadaciaaamcdsha0jadaciaaamccMcaPaqaiaiGaaaWiiadaciaaamcl6UinbqaiaiGaaaWiiadaciaaamcdEgaNnacaciaaamcBaaaleacaciaaamccWaGacaaaJaIXaqmcWaGacaaaJGGSaalcWaGacaaaJaIYaGmcWaGacaaaJGGSaalcWaGacaaaJaIZaWmaeqcaciaaamcaOGamaiGaaaWiiikaGIamaiGaaaWimiDaqNamaiGaaaWiiykaKcabGaGacaaaJGamaiGaaaWiSO7I0eabGaGacaaaJGamaiGaaaWim4zaC2aiaiGaaaWiSbaaSqaiaiGaaaWiiadaciaaamcdchaWjadaciaaamccYcaSiadaciaaamcdghaXjadaciaaamccYcaSiadaciaaamcdkhaYbqajaiGaaaWiaGccWaGacaaaJGGOaakcWaGacaaaJWG0baDcWaGacaaaJGGPaqkaeacaciaaamccWaGacaaaJaH7oaBdGaGacaaaJWgaaWcbGaGacaaaJGamaiGaaaWiGymaedabKaGacaaaJaakiadaciaaamccIcaOiadaciaaamcdsha0jadaciaaamccMcaPaqaiaiGaaaWiiadaciaaamceU7aSnacaciaaamcBaaaleacaciaaamccWaGacaaaJaIYaGmaeqcaciaaamcaOGamaiGaaaWiiikaGIamaiGaaaWimiDaqNamaiGaaaWiiykaKcabGaGacaaaJGamaiGaaaWiSO7I0eabGaGacaaaJGamaiGaaaWiSO7I0eabGaGacaaaJGamaiGaaaWiq4UdW2aiaiGaaaWiSbaaSqaiaiGaaaWiiadaciaaamcdMgaPbqajaiGaaaWiaGccWaGacaaaJGGOaakcWaGacaaaJWG0baDcWaGacaaaJGGPaqkaeacaciaaamccWaGacaaaJWIUlstaeacaciaaamccWaGacaaaJWIUlstaeacaciaaamccWaGacaaaJaH7oaBdGaGacaaaJWgaaWcbGaGacaaaJGamaiGaaaWimOta4eabKaGacaaaJaakiadaciaaamccIcaOiadaciaaamcdsha0jadaciaaamccMcaPaaacGaGacaaaJGLOaGaiaiGaaaWiyzkaaGaey4kaSYaaeWaaqaaceqaaiabew7aLnaaBaaaleaacqaIXaqmaeqaaOGaeiikaGIaemiDaqNaeiykaKcabaGaeqyTdu2aaSbaaSqaaiabikdaYaqabaGccqGGOaakcqWG0baDcqGGPaqkaeaacqWIUlstaeaacqWIUlstaeaacqaH1oqzdaWgaaWcbaGaemyAaKgabeaakiabcIcaOiabdsha0jabcMcaPaqaaiabl6Uinbqaaiabl6Uinbqaaiabew7aLnaaBaaaleaacqWGobGtaeqaaOGaeiikaGIaemiDaqNaeiykaKcaaiaawIcacaGLPaaacqGGUaGlcaWLjaGaaCzcaiabcIcaOiabigdaXiabikdaYiabcMcaPaaa@17D5@

In the dynamic equations in (12), the regulatory functions *g*_*p*_(*t*), *g*_*p*,*q*_(*t*) and *g*_*p*,*q*,*r*_(*t*) are shared by genes in the RGC. Therefore, the estimation of these functions from expression profiles *Y*_1_(*t*), *Y*_2_(*t*), …, *Y*_*N*_(*t*) can use also information from other genes to enhance our ability to reconstruct the *cis*-regulatory circuit of the gene of interest.

Finally, since the number of functions *g*_*p*_(*t*), *g*_*p*,*q*_(*t*), *g*_*p*,*q*,*r*_(*t*), … is finite, we can estimate these functions and the decay rates *λ*_1_(*t*), *λ*_2_(*t*), …, *λ*_*N*_(*t*) if the number *N *of dynamic equations in Equation (12) is large enough. Equation (12) can be rewritten in an algebraic form

X˜(t)=A˜(t)⋅G˜(t)+E(t),     (13)
 MathType@MTEF@5@5@+=feaafiart1ev1aaatCvAUfKttLearuWrP9MDH5MBPbIqV92AaeXatLxBI9gBaebbnrfifHhDYfgasaacH8akY=wiFfYdH8Gipec8Eeeu0xXdbba9frFj0=OqFfea0dXdd9vqai=hGuQ8kuc9pgc9s8qqaq=dirpe0xb9q8qiLsFr0=vr0=vr0dc8meaabaqaciGacaGaaeqabaqabeGadaaakeaaieaacuWFybawgaacaiabcIcaOiabdsha0jabcMcaPiabg2da9iqb=feabzaaiaGaeiikaGIaemiDaqNaeiykaKIaeyyXICTaf83raCKbaGaacqGGOaakcqWG0baDcqGGPaqkcqGHRaWkcqWFfbqrcqGGOaakcqWG0baDcqGGPaqkcqGGSaalcaWLjaGaaCzcaiabcIcaOiabigdaXiabiodaZiabcMcaPaaa@47BA@

where

X˜(t)=(Y˙1(t)Y˙2(t)⋮⋮Y˙i(t)⋮⋮Y˙N(t)),    G˜(t)=(g1(t)g2(t)⋮g1,2(t)⋮gp,q,r(t)λ1(t)λ2(t)⋮⋮λN(t)),    E(t)=(ε1(t)ε2(t)⋮⋮εi(t)⋮⋮εN(t)).
 MathType@MTEF@5@5@+=feaafiart1ev1aaatCvAUfKttLearuWrP9MDH5MBPbIqV92AaeXatLxBI9gBaebbnrfifHhDYfgasaacH8akY=wiFfYdH8Gipec8Eeeu0xXdbba9frFj0=OqFfea0dXdd9vqai=hGuQ8kuc9pgc9s8qqaq=dirpe0xb9q8qiLsFr0=vr0=vr0dc8meaabaqaciGacaGaaeqabaqabeGadaaakeaaieaacuWFybawgaacaiabcIcaOiabdsha0jabcMcaPiabg2da9maabmaabaqbaeqabqqaaaaabaqbaeqabqqaaaaabaGafmywaKLbaiaadaWgaaWcbaGaeGymaedabeaakiabcIcaOiabdsha0jabcMcaPaqaaiqbdMfazzaacaWaaSbaaSqaaiabikdaYaqabaGccqGGOaakcqWG0baDcqGGPaqkaeaacqWIUlstaeaacqWIUlstaaaabaGafmywaKLbaiaadaWgaaWcbaGaemyAaKgabeaakiabcIcaOiabdsha0jabcMcaPaqaauaabeqaceaaaeaacqWIUlstaeaacqWIUlstaaaabaGafmywaKLbaiaadaWgaaWcbaGaemOta4eabeaakiabcIcaOiabdsha0jabcMcaPaaaaiaawIcacaGLPaaacqGGSaalcaaMc8UaaGPaVlaaykW7caaMc8Uafe4raCKbaGaacqGGOaakcqWG0baDcqGGPaqkcqGH9aqpdaqadaqaauaabeqaeeaaaaqaauaabeqaeeaaaaqaauaabeqadeaaaeaacqWGNbWzdaWgaaWcbaGaeGymaedabeaakiabcIcaOiabdsha0jabcMcaPaqaaiabdEgaNnaaBaaaleaacqaIYaGmaeqaaOGaeiikaGIaemiDaqNaeiykaKcabaGaeSO7I0eaaaqaaiabdEgaNnaaBaaaleaacqaIXaqmcqGGSaalcqaIYaGmaeqaaOGaeiikaGIaemiDaqNaeiykaKcabaGaeSO7I0eabaGaem4zaC2aaSbaaSqaaiabdchaWjabcYcaSiabdghaXjabcYcaSiabdkhaYbqabaGccqGGOaakcqWG0baDcqGGPaqkaaaabaGaeq4UdW2aaSbaaSqaaiabigdaXaqabaGccqGGOaakcqWG0baDcqGGPaqkaeaacqaH7oaBdaWgaaWcbaGaeGOmaidabeaakiabcIcaOiabdsha0jabcMcaPaqaauaabeqadeaaaeaacqWIUlstaeaacqWIUlstaeaacqaH7oaBdaWgaaWcbaGaemOta4eabeaakiabcIcaOiabdsha0jabcMcaPaaaaaaacaGLOaGaayzkaaGaeiilaWIaaGPaVlaaykW7caaMc8UaaGPaVlabbweafjabcIcaOiabdsha0jabcMcaPiabg2da9maabmaabaqbaeqabqqaaaaabaqbaeqabqqaaaaabaGaeqyTdu2aaSbaaSqaaiabigdaXaqabaGccqGGOaakcqWG0baDcqGGPaqkaeaacqaH1oqzdaWgaaWcbaGaeGOmaidabeaakiabcIcaOiabdsha0jabcMcaPaqaaiabl6Uinbqaaiabl6UinbaaaeaacqaH1oqzdaWgaaWcbaGaemyAaKgabeaakiabcIcaOiabdsha0jabcMcaPaqaaiabl6UinbqaauaabeqaceaaaeaacqWIUlstaeaacqaH1oqzdaWgaaWcbaGaemOta4eabeaakiabcIcaOiabdsha0jabcMcaPaaaaaaacaGLOaGaayzkaaGaeiOla4caaa@C6D3@

Then, we have the following dynamic equations for all time profiles

(
														X˜(t1)
														 X˜(t2)⋮
														  X˜(tm))=(
														  A˜(t1)0↔00
													A˜(t2)↕↕⋱00↔0
														   A˜(tm))·(
													G˜(t1)
														  G˜(t2)⋮
														 G˜(tm))+(E(t1)E(t2)⋮E(tm)).     (14)
 MathType@MTEF@5@5@+=feaafiart1ev1aaatCvAUfKttLearuWrP9MDH5MBPbIqV92AaeXatLxBI9gBaebbnrfifHhDYfgasaacH8akY=wiFfYdH8Gipec8Eeeu0xXdbba9frFj0=OqFfea0dXdd9vqai=hGuQ8kuc9pgc9s8qqaq=dirpe0xb9q8qiLsFr0=vr0=vr0dc8meaabaqaciGacaGaaeqabaqabeGadaaakeaadaqadaqaauaabeqaeeaaaaqaaiqbbIfayzaaiaGaeiikaGIaemiDaq3aaSbaaSqaaiabigdaXaqabaGccqGGPaqkaeaacuqGybawgaacaiabcIcaOiabdsha0naaBaaaleaacqaIYaGmaeqaaOGaeiykaKcabaGaeSO7I0eabaGafeiwaGLbaGaacqGGOaakcqWG0baDdaWgaaWcbaGaemyBa0gabeaakiabcMcaPaaaaiaawIcacaGLPaaacqGH9aqpdaqadaqaauaabeqaeqaaaaaabaGafeyqaeKbaGaacqGGOaakcqWG0baDdaWgaaWcbaGaeGymaedabeaakiabcMcaPaqaaiabicdaWaqaaiabgsziRcqaaiabicdaWaqaaiabicdaWaqaaiqbbgeabzaaiaGaeiikaGIaemiDaq3aaSbaaSqaaiabikdaYaqabaGccqGGPaqkaeaaaeaacqWIvgIyaeaacqWIvgIyaeaaaeaacqWIXlYtaeaacqaIWaamaeaacqaIWaamaeaacqGHugYQaeaacqaIWaamaeaacuqGbbqqgaacaiabcIcaOiabdsha0naaBaaaleaacqWGTbqBaeqaaOGaeiykaKcaaaGaayjkaiaawMcaaiabl+y6NnaabmaabaqbaeqabqqaaaaabaGafe4raCKbaGaacqGGOaakcqWG0baDdaWgaaWcbaGaeGymaedabeaakiabcMcaPaqaaiqbbEeahzaaiaGaeiikaGIaemiDaq3aaSbaaSqaaiabikdaYaqabaGccqGGPaqkaeaacqWIUlstaeaacuqGhbWrgaacaiabcIcaOiabdsha0naaBaaaleaacqWGTbqBaeqaaOGaeiykaKcaaaGaayjkaiaawMcaaiabgUcaRmaabmaabaqbaeqabqqaaaaabaGaeeyrauKaeiikaGIaemiDaq3aaSbaaSqaaiabigdaXaqabaGccqGGPaqkaeaacqqGfbqrcqGGOaakcqWG0baDdaWgaaWcbaGaeGOmaidabeaakiabcMcaPaqaaiabl6UinbqaaiabbweafjabcIcaOiabdsha0naaBaaaleaacqWGTbqBaeqaaOGaeiykaKcaaaGaayjkaiaawMcaaiabc6caUiaaxMaacaWLjaGaeiikaGIaeGymaeJaeGinaqJaeiykaKcaaa@94B1@

Let us denote the above equations in the following simple algebraic form


								 X˜=Φ˜⋅Θ˜+M.     (15)
 MathType@MTEF@5@5@+=feaafiart1ev1aaatCvAUfKttLearuWrP9MDH5MBPbIqV92AaeXatLxBI9gBaebbnrfifHhDYfgasaacH8akY=wiFfYdH8Gipec8Eeeu0xXdbba9frFj0=OqFfea0dXdd9vqai=hGuQ8kuc9pgc9s8qqaq=dirpe0xb9q8qiLsFr0=vr0=vr0dc8meaabaqaciGacaGaaeqabaqabeGadaaakeaacuqGybawgaacaiabg2da9iqbfA6agzaaiaGaeyyXICTafuiMdeLbaGaacqGHRaWkcqqGnbqtcqqGUaGlcaWLjaGaaCzcaiabbIcaOiabbgdaXiabbwda1iabbMcaPaaa@3C07@

In Equation (15), 
							X˜
 MathType@MTEF@5@5@+=feaafiart1ev1aaatCvAUfKttLearuWrP9MDH5MBPbIqV92AaeXatLxBI9gBaebbnrfifHhDYfgasaacH8akY=wiFfYdH8Gipec8Eeeu0xXdbba9frFj0=OqFfea0dXdd9vqai=hGuQ8kuc9pgc9s8qqaq=dirpe0xb9q8qiLsFr0=vr0=vr0dc8meaabaqaciGacaGaaeqabaqabeGadaaakeaacuqGybawgaacaaaa@2DF4@ and Φ˜
 MathType@MTEF@5@5@+=feaafiart1ev1aaatCvAUfKttLearuWrP9MDH5MBPbIqV92AaeXatLxBI9gBaebbnrfifHhDYfgasaacH8akY=wiFfYdH8Gipec8Eeeu0xXdbba9frFj0=OqFfea0dXdd9vqai=hGuQ8kuc9pgc9s8qqaq=dirpe0xb9q8qiLsFr0=vr0=vr0dc8meaabaqaciGacaGaaeqabaqabeGadaaakeaacuqHMoGrgaacaaaa@2E37@ can be calculated from microarray data for the RGC of the gene of interest, which can then be used to estimate the vector Θ˜^
 MathType@MTEF@5@5@+=feaafiart1ev1aaatCvAUfKttLearuWrP9MDH5MBPbIqV92AaeXatLxBI9gBaebbnrfifHhDYfgasaacH8akY=wiFfYdH8Gipec8Eeeu0xXdbba9frFj0=OqFfea0dXdd9vqai=hGuQ8kuc9pgc9s8qqaq=dirpe0xb9q8qiLsFr0=vr0=vr0dc8meaabaqaciGacaGaaeqabaqabeGadaaakeaacuqHyoqugaacgaqcaaaa@2E43@ by the least squares method, leading to the following solution:

Θ˜^=(Φ˜TΦ˜)−1Φ˜
								X˜.     (16)
 MathType@MTEF@5@5@+=feaafiart1ev1aaatCvAUfKttLearuWrP9MDH5MBPbIqV92AaeXatLxBI9gBaebbnrfifHhDYfgasaacH8akY=wiFfYdH8Gipec8Eeeu0xXdbba9frFj0=OqFfea0dXdd9vqai=hGuQ8kuc9pgc9s8qqaq=dirpe0xb9q8qiLsFr0=vr0=vr0dc8meaabaqaciGacaGaaeqabaqabeGadaaakeaacuqHyoqugaacgaqcaiabg2da9iabcIcaOiqbfA6agzaaiaWaaWbaaSqabeaacqWGubavaaGccuqHMoGrgaacaiabcMcaPmaaCaaaleqabaGaeyOeI0IaeGymaedaaOGafuOPdyKbaGaacuqGybawgaacaiabc6caUiaaxMaacaWLjaGaeiikaGIaeGymaeJaeGOnayJaeiykaKcaaa@401C@

After Θ˜^
 MathType@MTEF@5@5@+=feaafiart1ev1aaatCvAUfKttLearuWrP9MDH5MBPbIqV92AaeXatLxBI9gBaebbnrfifHhDYfgasaacH8akY=wiFfYdH8Gipec8Eeeu0xXdbba9frFj0=OqFfea0dXdd9vqai=hGuQ8kuc9pgc9s8qqaq=dirpe0xb9q8qiLsFr0=vr0=vr0dc8meaabaqaciGacaGaaeqabaqabeGadaaakeaacuqHyoqugaacgaqcaaaa@2E43@ is estimated from Equation (16), the regulatory functions 
								G˜(t)
 MathType@MTEF@5@5@+=feaafiart1ev1aaatCvAUfKttLearuWrP9MDH5MBPbIqV92AaeXatLxBI9gBaebbnrfifHhDYfgasaacH8akY=wiFfYdH8Gipec8Eeeu0xXdbba9frFj0=OqFfea0dXdd9vqai=hGuQ8kuc9pgc9s8qqaq=dirpe0xb9q8qiLsFr0=vr0=vr0dc8meaabaqaciGacaGaaeqabaqabeGadaaakeaacuqGhbWrgaacaiabcIcaOiabdsha0jabcMcaPaaa@30F5@ in Equation (13) can be reconstructed for the genes in the RGC at every time point. However, in Equation (12), the degradation rate *λ*(*t*) is a time-varying function and is affected by both the error terms and experimental data. Therefore, in order to reduce the influence on degradation rate, we simplify Equation (12) and average the negative gradients of *λ*(*t*) to obtain the constant value λ^
 MathType@MTEF@5@5@+=feaafiart1ev1aaatCvAUfKttLearuWrP9MDH5MBPbIqV92AaeXatLxBI9gBaebbnrfifHhDYfgasaacH8akY=wiFfYdH8Gipec8Eeeu0xXdbba9frFj0=OqFfea0dXdd9vqai=hGuQ8kuc9pgc9s8qqaq=dirpe0xb9q8qiLsFr0=vr0=vr0dc8meaabaqaciGacaGaaeqabaqabeGadaaakeaacuaH7oaBgaqcaaaa@2E72@. Then the estimated λ^
 MathType@MTEF@5@5@+=feaafiart1ev1aaatCvAUfKttLearuWrP9MDH5MBPbIqV92AaeXatLxBI9gBaebbnrfifHhDYfgasaacH8akY=wiFfYdH8Gipec8Eeeu0xXdbba9frFj0=OqFfea0dXdd9vqai=hGuQ8kuc9pgc9s8qqaq=dirpe0xb9q8qiLsFr0=vr0=vr0dc8meaabaqaciGacaGaaeqabaqabeGadaaakeaacuaH7oaBgaqcaaaa@2E72@ is substituted into Equation (7) to re-identify the *cis*-regulatory functions to derive the final regulatory functions. Using this procedure, we can avoid the effects of the time-varying function *λ*(*t*) on the identification process and reduce the influence by different experimental data. Hence, the degradation rate can be estimated. After the functions are estimated, they can be plugged into Equation (11) and then the reconstruction of the *cis*-regulatory circuit of the gene of interest is completed.

## Authors' contributions

L.H. Lin carried out the model design and computation of this study, and drafted the manuscript. H.C. Lee participated in the design of the study and drafted the manuscript. W.H. Li amended and improved the design and the presentation of the study. B.S. Chen gave the topic and suggestions and was responsible for the entire study. All authors read and approved the final manuscript.

## Supplementary Material

Additional File 1 The cell cycle genes and their connectivities to *cis *elements. 769 cell cycle genes defined by Spellman *et al. *[18] were selected from a total of 6178 genes in the data set. "1" denotes the connection of a *cis *element with a gene, while "0" means no connection. The main *cis *element data were compiled from the data set of Simon *et al. *2001 [4] by choosing a *P *value (significance level) ≤ 0.0015. Under this threshold, many interactions among *cis *elements for genes confirmed by the conventional data are included [35,36]. Additionally, we modified some *cis *element data, using well-known experimental information to correct false negatives [35,36]. Click here for file
